# Supporting the transition from weight loss to maintenance: development and optimisation of a face-to-face behavioural intervention component

**DOI:** 10.1080/21642850.2016.1269233

**Published:** 2017-01-06

**Authors:** Kirby Sainsbury, Claire L. Cleland, Elizabeth H. Evans, Ashley Adamson, Alan Batterham, Stephan U. Dombrowski, Paul Gellert, Moira Hill, Dominika Kwasnicka, Dawn Scott, Falko F. Sniehotta, Martin White, Vera Araújo-Soares

**Affiliations:** ^a^ Institute of Health & Society, Faculty of Medical Sciences, Newcastle University, Newcastle Upon Tyne, UK; ^b^ Fuse, The UKCRC Centre for Translational Research in Public Health, UK; ^c^ School of Planning, Architecture and Civil Engineering, Queen’s University Belfast, Belfast, UK; ^d^ Division of Psychology, School of Natural Sciences, University of Stirling, Stirling, UK; ^e^ Newcastle upon Tyne Hospitals NHS Foundation Trust, Newcastle upon Tyne, UK; ^f^ Charité – Universitätsmedizin Berlin, CC1 – Institut für Medizinische Soziologie, Berlin, Germany; ^g^ Newcastle upon Tyne City Council, Public Health, Civic Centre, Newcastle upon Tyne, UK; ^h^ Health and Social Care Institute, Teesside University, Middlesbrough, UK; ^i^ CEDAR, UKCRC Centre for Diet and Activity Research and MRC Epidemiology Unit, University of Cambridge School of Clinical Medicine, Cambridge, UK; ^j^ Human Nutrition Research Centre, Institute of Health & Society, Newcastle University, Newcastle upon Tyne, UK

**Keywords:** Weight loss maintenance, obesity, self-regulation, intervention development, acceptability

## Abstract

After weight loss, most individuals regain lost weight. Interventions to support the transition from successful loss to weight loss maintenance (WLM), regardless of the method of prior weight loss, are needed. The aims of this study were to (1) develop a face-to-face behavioural intervention session to support overweight and obese individuals who have recently lost a clinically significant amount of weight in the transition to WLM; (2) to assess the single-session intervention for acceptability and feasibility prior to its use in a larger, 12-month, multi-component trial; and (3) to optimise the intervention session for future use based on participant feedback. Participants with a Body Mass Index of ≥25 kg/m^2^ prior to a ≥5% weight loss in the previous 12 months were recruited via the local government authority and community-based advertisements. Each attended the one-hour session with a trained facilitator, which focused on setting maintenance-relevant weight, eating, and physical activity goals. Semi-structured interviews were carried out immediately post-session to obtain feedback on the acceptability of this intervention component. Data were used to generate recommendations for changes to the session, which were discussed by the team, and used to optimise the session. Seventeen participants (13 female; median WL = 13%) were recruited. All participants evaluated the intervention session positively; 11 participants suggested improvements including reducing information provision in favour of greater focus on identifying and coping with barriers, and the inclusion of practical examples. The systematic refinement and optimisation process resulted in an acceptable and feasible face-to-face behavioural intervention session (described here), which will be tested as part of a multi-component intervention. We anticipate the session could be used to supplement existing support including online services, and has the potential to benefit people who have lost a clinically significant amount of weight to achieve WLM over the long term.

## 1. Introduction

The majority of adults in the United Kingdom (UK) are overweight or obese – in 2013, 67% of men and 57% of women were classified as such (Health and Social Care Information Centre, 2013). These figures are higher than most developed countries and represent the highest prevalence of overweight and obesity in Europe (Health and Social Care Information Centre, 2013; Organisation for Economic Co-operation and Development, 2012). Obesity is a leading cause of premature morbidity and mortality (Adams et al., 2006; Must et al., 1999), with overweight or obese individuals having an increased likelihood of developing chronic, life-threatening conditions such as cancer, type-2-diabetes, and cardiovascular disease (Adams et al., 2006; Calle, Rodriguez, Walker-Thurmond, & Thun, 2003; Mokdad, Bales, Greenlund, & Mensah, 2003; Must et al., 1999; Poirier et al., 2006). Furthermore, the implications of overweight/obesity reach wider than the health of the individual, with the Department of Health reporting that the direct costs for the UK National Health Service associated with obesity alone were estimated to be £5.1 billion per year (2011). Behaviour change interventions that focus on supporting individuals to make changes to their dietary behaviours are effective in reducing weight and improving health (Dombrowski, Avenell, & Sniehotta, 2010; Leventhal, Weinman, Leventhal, & Phillips, 2008; Tsai & Wadden, 2005). However, despite initial success in terms of weight loss, most individuals regain approximately half the weight they lost within one year, and the rest of the lost weight within three to five years (Avenell et al., 2004; Curioni & Lourenco, 2005).

Weight loss maintenance (WLM) is defined as a process of maintaining a nominally significant intentional weight loss accomplished by one’s own efforts (Elfhag & Rössner, 2005). Specifically, weight loss of 5–10% is deemed clinically significant (Crawford, Jeffery, & French, 2000; Wing & Hill, 2001), as it is associated with reduced obesity-related morbidity and mortality (Franz et al., 2007); and maintenance is defined as keeping this weight off for a minimum of six months (Elfhag & Rössner, 2005) to one year (Wing & Hill, 2001). While some health benefits might be achieved and maintained even if weight is subsequently regained (e.g., diabetes prevention; Diabetes Prevention Program Research Group, 2009), interventions that support sustained WLM are needed to optimise the long-term effects of successful weight loss interventions (Penn et al., 2013). Evidence has shown that it is possible to decrease the levels of weight regain following initial weight loss (Simpson, Shaw, & McNamara, 2011; Turk et al., 2009). For example, in a systematic review of 11 randomised controlled trials (RCTs), participants who received extended care interventions were less likely to regain lost weight compared to control groups (Middleton, Patidar, & Perri, 2012). Another systematic review of WLM RCTs found a difference in weight regain over 12 months of 1.6 kg between participants who had received intervention and those who had not (Dombrowski, Knittle, Avenell, Araújo-Soares, & Sniehotta, 2014b).

In reality, individuals attempt to lose weight using a multitude of different supported and unsupported methods. WLM on the other hand, involves a series of complex behaviour and goal-related changes (i.e., from initiation to maintenance) that are often associated with the transition from an energy deficit diet to a sustainable WLM lifestyle (Sniehotta, Simpson, & Greaves, 2014). Combined with the finding that effective strategies for weight loss and maintenance differ (Sciamanna et al., 2011), the implication of this is a need to develop flexible WLM interventions that are applicable independently of prior weight loss method. In contrast, inducing weight loss prior to randomisation to WLM conditions inevitably results in a more homogeneous sample than would be obtained if the former type of participant were recruited, with the effectiveness of the WLM intervention therefore being closely linked to the effectiveness and suitability of the weight loss phase. Despite this important methodological consideration, only 3 of the 45 included studies in a WLM systematic review recruited individuals who had lost clinically significant amounts of weight *on their own* before the WLM intervention was offered (Dombrowski et al., 2014b).

Based on the clear need to fill this gap, here we describe the first step in the development of a multi-component behavioural WLM intervention; namely, a single face-to-face session with a trained facilitator, which was designed to be attended at the commencement of a broader, 12-month duration WLM programme. Briefly, the NULevel intervention is a primarily SMS-delivered, self-regulatory intervention consisting of a face-to-face session, followed by frequent text messages to prompt self-weighing (using study scales with weights transmitted digitally to the study team) and weekly diary completion (including achievement of eating and physical activity goals), and to deliver theory-based behaviour change techniques (BCTs) targeting motivation, psychological resources, social support, and managing conflicting priorities, among other themes. The entire intervention has been described elsewhere (Evans et al., 2015) and is being tested for effectiveness in an RCT design with an internal pilot study and process evaluation to further establish acceptability and feasibility. Outside of this specific context, it is anticipated that the intervention session could also be used as a supplement to other existing weight management programmes to aid the transition from weight loss to WLM, regardless of initial weight loss method. The aims of this study were to (1) develop the content of the face-to-face component of the behavioural WLM intervention; (2) establish the acceptability and feasibility of this novel WLM intervention component; and (3) optimise the intervention session in line with participant feedback.

## 2. Methods

### 2.1. Study design

Interventions should be based on best available evidence and theory to maximise effectiveness and understand how and why interventions do or do not work (Bartholomew, Parcel, Kok, Gottlieb, & Fernandez, 2011; Craig et al., 2008; Hoffmann et al., 2014; Michie & Abraham, 2004). Consistent with the recommendations of the Medical Research Council and Reach Effectiveness Adoption Implementation Maintenance (RE-AIM) frameworks for the development and evaluation of complex interventions (Craig et al., 2008; Glasgow, McKay, Piette, & Reynolds, 2001), as well as the Template for Intervention Description and Replication (TIDieR) checklist for reporting interventions (Hoffmann et al., 2014), the development, piloting, and optimisation process of the intervention in the target population is described here. Specifically, this involved the delivery of the initial version of the intervention session to members of the target population, followed by user engagement research to elicit feedback directly after the session, and the feedback was used to revise and optimise the intervention session for future use.

### 2.2. Participant recruitment procedure

Participants were recruited using two routes. First, individuals participating in a weight loss programme funded by the local government authority were invited to participate on behalf of the study team. Second, individuals were recruited via advertisements (posters, e-posters, or the university intranet). All individuals who contacted the research team were provided with study information and given the opportunity to ask questions prior to consent. Those interested in participating were screened for study inclusion criteria: (i) intentional loss of at least 5% of body weight in the previous 12 months; and (ii) a pre-weight loss Body Mass Index (BMI) of ≥25 kg/m^2^ (adjusted appropriately for participants from Asian backgrounds; Jafar, Chatuverdi, & Pappas, 2006). Exclusion criteria included recent weight gain due to pregnancy, and unintentional weight loss (e.g., through illness).

Individuals meeting the inclusion criteria were posted a pre-session pack approximately one week prior to the face-to-face intervention session. This included the participant information sheet, a four-day food and activity diary, and a pedometer (Omron Walking Style II, piezoelectric pedometer-Omron Healthcare Ltd. Milton Keynes, UK). Prior to attending the face-to-face intervention session, participants recorded their food and fluid intake, portion size, and any other additional detail perceived as relevant (e.g., cooking methods, context) in a provided diary. In addition, participants self-monitored their daily steps using the pedometer and recorded them in the diary (BCT: prompt self-monitoring of behaviour – eating and activity). They then attended a face-to-face session at the university campus at a convenient time (between July and September 2013). Participants were asked to bring their completed pre-session pack to the intervention session.

### 2.3. Baseline measures

Socio-demographic and weight measures were collected at the beginning of the face-to-face session including sex, occupation, current weight, and current height. Weight was self-reported rather than measured objectively because the goal of this early stage of the wider study was primarily to assess acceptability of this intervention component rather than its effectiveness for WLM. Data were also gathered on weight management goals (i.e., to lose or to maintain weight), ideal weight, amount of weight lost in their most successful weight loss attempt (self-report), number and duration of weight loss attempts in the past 12 months, and the weight loss strategies employed in the last attempt (see Table 1 for a list of strategies).

**Table 1.** Content description of the optimised intervention session.

Section	Content	BCTs
Introduction	Welcome and consent; reiterate study background; explain purpose and format of the session	
1. Weight	Review weight history and methods of weight loss used in most recent attempt^a^	Prompt focus on past success
	Agree overall weight maintenance goal (green zone) and regain thresholds for red and yellow zones using the traffic light system	Goal setting (outcome)
	Explain the rationale for frequent self-monitoring of weight and how to make subtle changes to behaviour if weight is starting to trend upwards; encourage participants to monitor their weight	Prompt self-monitoring of behavioural outcome
2. Eating	Explain concept of ‘trigger foods’; identification of personal trigger foods/drinks and typical decision and behaviour around these foods; generate a list of potential food swaps to replace these foods	Barrier identification Problem-solving Relapse prevention/coping planning
	Explain concept of challenging situations; identification of personal challenging situations and typical decision making and behaviour in these situations; generate a coping plan for how to manage such situations	Barrier identification Problem-solving Relapse prevention/coping planning
	Briefly review eating behaviours using four-day food diary: habits, frequency of eating; nutritional adequacy; context; any uncontrolled eating; whether participant desires any dietary changes. If a participant is not currently happy with their eating plan and/or does not feel that it is sustainable: outline three alternate eating plans and discuss how the chosen one could be implemented. These tasks are combined with the goal-setting task below (used to generate ideas for SMART goals)	Provide feedback on performance Provide instruction on how to perform the behaviour Provide information on where and when to perform the behaviour
	Provide the rationale for goal setting using a SMART goal framework and coping planning; identify and formulate 2 SMART eating goals for WLM – generate a detailed and specific plan for how to achieve behavioural goals (how, where, and when behaviour will be performed, and potential sources of social support), as well as identifying anticipated barriers to enacting goal, and possible solutions to overcome these barriers	Goal-setting (behaviour) Problem-solving Action planning Coping planning Plan social support or social change
	Reiterate the rationale for regular self-monitoring and encourage participants to continue monitoring their progress towards their behavioural goals	Prompt self-monitoring of behaviour
	Encourage participants to review and revise their goals and action and coping plans in light of progress towards their goals	Prompt review of behavioural goals
3. Physical activity	Highlight the importance of physical activity for WLM and overall health, focusing on any personal barriers to (e.g., injury), or health consequences of (e.g., reduced cardiovascular risk), regular physical activity	Provide information on consequences of behaviour in general Provide information on consequences of behaviour to the individual
	Review and discuss current physical activity levels (based on step counts and any additional activity) including any personal barriers to being more active	Provide feedback on performance Barrier identification
	Identify and formulate a SMART goal for increasing physical activity (usually a daily step goal) – generate a detailed and specific plan for how to achieve behavioural goals (how, where, and when behaviour will be performed, and potential sources of social support), as well as coping planning in order to overcome any experienced or anticipated barriers to goal achievement. Refer back to the volitional help sheet that was completed as part of the pre-session questionnaire to aid with identifying barriers and generating potential solutions	Goal-setting (behaviour) Action planning Barrier identification Coping planning Plan social support or social change
	Reiterate the rationale for regular self-monitoring and reviewing goals and plans in line with progress	Prompt self-monitoring of behaviour Prompt review of behavioural goals
Summary	Summarise the participant’s weight maintenance, eating, and activity goals; reiterate importance of regular self-monitoring of behaviour and outcome in order to achieve those goals; answer any questions	

Note: WLM: weight loss maintenance; SMART: specific, measureable, achievable, relevant, time-limited.

^a^List of weight loss strategies included: reduced amount of food; increased fruit and vegetable consumption; reduced fat consumption; increased/started physical activity; switched to foods with lower calories; reduced sugar, chocolates, or sweets; reduced junk food/fast food; changed eating habits/patterns; increased water consumption; joined a weight loss programme; ate ‘diet’ food products; took weight loss pills (prescription or non-prescription) or herbs/supplements; followed a special diet; skipped meals; used a liquid diet formula/very low calorie diet.

### 2.4. Intervention description

The development of the face-to-face session specifically and the broader 12-month, multi-component intervention more generally (Evans et al., 2015), were informed by several trials and systematic reviews, which together provided evidence for the relevance of self-regulation (Dombrowski, Knittle, Avenell, Araújo-Soares, & Sniehotta, 2014a; Dombrowski et al., 2014b; Teixeira et al., 2015), self-efficacy (Linde, Rothman, Baldwin, & Jeffery, 2006; Shin et al., 2011; Wilson, Fabio, Hill, Wen, & Estabrooks, 2015), and relapse prevention in maintenance of behaviour change generally (Kwasnicka, Dombrowski, White, & Sniehotta, 2016) and specifically in WLM (Latner, McLeod, O'Brien, & Johnston, 2013). The intervention content and materials were developed by a team of registered dietitians, nutritionists, and behavioural scientists with expertise and experience in designing and delivering weight management programmes. In addition, a patient and public involvement panel of six adults who had experience with weight loss, and two lay members of the trial steering committee with similar experience, were consulted in the development process. Materials were additionally informed by a publically available resource designed for a previous weight loss RCT (Sniehotta et al., 2005, 2011).

For the purposes of this study, the initial version of the face-to-face intervention session was delivered in a single, one-on-one session by a trained facilitator (dietitian), in a location at the university. It consisted of five main sections:

1. Setting a weight maintenance goal (BCT: goal setting – outcome): given that the full intervention was designed to aid WLM, the primary goal was to avoid regain (i.e., stay at current weight), thereby safeguarding the progress that participants had already made in losing weight. In addition to this goal (classified as the ‘green zone’), participants were prompted to set regain thresholds using a traffic light system for weight maintenance (Wing, Tate, Gorin, Raynor, & Fava, 2006) – specifying ‘yellow’ and ‘red’ zones, which correspond to approximately 2.5% and 5% regain respectively and represent weights that the participant would prefer not to exceed and would signal the need to make changes to their behaviour in order to prevent further regain and lose any regained weight. Participants could also set a goal for further weight loss, although it was stressed that maintenance rather than additional weight loss was the focus of the intervention. They were then encouraged to frequently monitor their weight following the session in order to detect weight regain (BCT: prompt self-monitoring of behaviour; note, the facilitator did not specify the frequency of weighing).
2. Reviewing the participant’s weight loss history, including the strategies used to lose weight most recently and in the past. The main purpose of this section was to boost self-efficacy (BCT: focus on their past success), as well as to encourage the continued use of personally effective strategies where these were considered sustainable and conducive to general health.
3. Reviewing the participants’ food diary and using this to prompt discussion of their eating patterns and routines, the nutritional adequacy of the current diet (e.g., fruit and vegetable consumption), the context of eating (social, physical, and emotional), and any antecedents and/or consequences of eating that are counter-productive to weight management. The facilitator then provided personalised feedback (BCT: provide feedback on performance), tailored according to the information provided in the diary and the weight goals of each participant. In negotiation with the participant, they then agreed on a number of ways to improve their diet (BCT: goal setting – behaviour), while also emphasising the need for maintenance of effective healthy strategies and food choices that the participant was already using. Specific suggestions for dietary changes were taken from three alternate plans, and participants were provided with written materials to support these choices. For the purposes of this study, the options were a calorie-controlled diet (Finer, 2001), the Mediterranean diet (Esposito, Kastorini, Panagiotakos, & Giugliano, 2011), and Change4Life (a UK government issued weight management programme; Department of Health, 2009). Given the limited evidence for the effectiveness of any one dietary plan over another (Jolly et al., 2010), these could be replaced with other options. Indeed, the available evidence suggests that most diet plans can be effective in achieving significant weight loss if adherence is good (Pagoto & Appelhans, 2013).
4. Relapse prevention/coping planning (BCT):
  - Identifying ‘trigger foods’ (defined as foods that the participant finds difficult to limit consumption of and therefore place them at risk of over-eating; e.g., chocolate) and generating ideas for healthier alternatives.
  - Identifying tempting situations (e.g., socialising) and generating ideas for how to manage a healthy eating plan while continuing to engage in these situations so that they do not lead to over-consumption.
5. Reviewing participants’ current level of physical activity (i.e., step counts and any other activity) and comparing their activity to the UK national recommendations for adults (75 minutes of vigorous or 150 minutes of moderate physical activity per week (Bull, 2010), which equates to approximately 10,000 steps per day (National Health Service Choices, 2014); BCT: provide feedback on performance).

Notes from all the tasks were recorded by the facilitator in a purposely designed study booklet (see Supplementary File 1), which participants were given to take away with them at the end of the session. The session concluded with the facilitator summarising the agreed goals to check participant understanding and to reinforce their plans for WLM.

### 2.5. Post-intervention interview

Immediately following the face-to-face intervention session, a semi-structured interview was conducted by an independent researcher. The interview followed a standardised topic guide based on a previous acceptability and feasibility study of a behavioural intervention for obese participants (Dombrowski, Sniehotta, Johnston, et al., 2012). Specifically, participants were asked to comment on (1) the parts of the session that they liked the best (with prompts to reflect on the weight goal-setting task, information on diet and physical activity, and the two relapse prevention tasks); (2) the structure and delivery of the session; (3) the session materials; (4) suggestions for improvement; and (5) general comments. The interview responses were captured using pre-specified record sheets and real-time note taking. Interviews lasted a median of 17 minutes (inter-quartile range (IQR) = 15–18 minutes). Participants were also asked to rate the perceived usefulness and understanding of the session using a Likert scale ranging from 1 (not useful/not understandable at all) to 10 (extremely useful/understandable).

### 2.6. Analysis

Participant socio-demographic data and quantitative acceptability ratings, as assessed in the post-session interview, were analysed descriptively using median and IQRs. The qualitative data collected during the semi-structured interviews was organised into relevant categories for intervention refinement – this was initially done by one team member and then discussed in detail with another team member; any disagreements were resolved through discussion. Pre-specified categories that were used in this process included: (1) pre-session procedures; (2) intervention session content and procedures; and (3) intervention session materials.

The feasibility of the intervention procedures and materials were assessed by recording and reporting face-to-face session length (timed by the intervention facilitator), assessing the appropriateness of the intervention setting, and participant adherence to pre-session tasks (i.e., completion of food diary, use of pedometer).

### 2.7. Refinement and optimisation of the face-to-face WLM intervention

The optimisation process used a sequential approach. Based on the interview data and agreed upon categories for intervention refinement (completed by two team members), each member of the intervention development team was given the opportunity to submit recommendations for changes to the content, delivery and structure, or materials via email. All recommendations were collated by one team member and circulated back to the team, and then discussed in a consensus conference. Discussions were structured around each specific recommendation and drew on the team’s combined expertise across a range of disciplines, which included dietetics and human nutrition, behavioural science, health psychology, intervention development, public health, and physical activity. Subsequently, suggested changes that were in line with empirical evidence and deemed feasible by the delivery team were made to the intervention session protocol in order to optimise the content, materials, and procedures. All changes and their concomitant rationales were similarly organised and recorded under the optimisation categories pre-session procedures, intervention session content and procedures, and intervention session materials.

## 3. Results

### 3.1. Participants

Seventeen participants (13 women) took part in the study. Prior to initiating weight loss, the median BMI for the women was 33.1 kg/m^2^ (IQR = 28.7–38.3; weight: median = 87.1 kg; IQR = 79.2–98.6), with nine participants classified as obese and four as overweight. For the men, the median BMI, prior to losing weight, was lower, at 28.6 kg/m^2^ (IQR = 27.2–32.9; weight: median = 90.6 kg; IQR = 86.0–104.2); one man was classified as obese and three were overweight. Over the course of the preceding 12 months, median-reported weight loss for the women was 10 kg (IQR = 5.9–15.9), which equated to 12.4% of body weight (IQR = 7.0–18.4). The men had lost a median of 12.8 kg (IQR = 6.0–14.6), equivalent to 12.7% (IQR = 6.7–15.9).

At the time of the study, the median BMI of the women was 27.0 kg/m^2^ (IQR = 25.2–31.9; weight: median = 72.7 kg, IQR = 67.2–80.8), with two participants in the healthy weight range, eight overweight, and three remaining obese. For the men, the median BMI was 25.6 kg/m^2^ (IQR = 23.9–28.8; weight: median = 81.0 kg, IQR = 75.3–91.2); one was in the healthy weight range and three remained overweight.

Recent weight loss was achieved using various methods, most commonly reducing portion sizes (*n* = 13), reducing sugar, fat, and calorie consumption (all *n* = 11), reducing junk food (*n* = 10), increasing fruit and vegetable consumption (*n* = 13), and increasing the level of physical activity (*n* = 13).

### 3.2. Feasibility

The median delivery time of the intervention was 48 minutes (IQR = 45–63 minutes); no negative feedback was received regarding the setting of the session; all 17 participants adhered to pre-session tasks including completing their four-day food diary and recording their level of physical activity for the same period of time.

### 3.3. Acceptability

#### 3.3.1. Perceived understanding and usefulness

Immediately following the face-to-face session, the median usefulness of the intervention session was rated by participants as 8.5/10 (IQR = 8–9), and the median understanding was rated as 9.5/10 (IQR = 9–10).

#### 3.3.2. Feedback: immediate post-intervention

There was good agreement between the two team members who were responsible for categorisation of interview responses for intervention optimisation and refinement. Qualitative feedback after the session suggested that participants evaluated the intervention content and format positively. In general, participants found the content informative and interesting. Participants appreciated the tailored dietary advice and physical activity recommendations that were provided. They reported that this aspect of the intervention was motivating and helpful, prompting them to focus on specific changes they needed to make to successfully manage their weight. They also appreciated the constructive feedback on their current behaviour (based on food diaries and step counts completed prior to the session), reflecting that it was reassuring to know that they were making good choices a lot of the time already. Several participants reported that the two coping planning activities were useful in prompting them to consider specifically how they would go about implementing change (e.g., focusing on time management).

Regarding the format of the intervention, participants reported being satisfied; in particular mentioning the relaxed atmosphere, clear structure, and ease of understanding. Similarly, positive feedback on the materials was obtained – participants felt the materials were attractive, well laid out, and easy to follow, and they appreciated the opportunity to take content away with them to refer back to at a later time. Several participants stated that the process of completing a four-day food diary was useful in drawing their attention to their eating, and prompting change even before attending the session.

#### 3.3.3. Proposed improvements

Whilst six participants felt that no changes were required to the intervention, the remaining 11 suggested some improvements, either directly or by inference. All suggestions were considered in the intervention refinement and optimisation process. Suggestions included that the intervention was too focused on providing information, most of which was already known. Instead, it was suggested to assess participants’ prior knowledge, and have a greater emphasis on identifying the participant’s current areas of difficulty and generating ideas to overcome these (e.g., emphasising the importance of self-regulatory and relapse prevention strategies). One participant felt that greater acknowledgement of the ‘real world’ barriers that prevent people from following their ideal weight management plan would be useful, and suggested an explicit discussion of psychological barriers, specifically emotional eating and motivational factors.

Several participants reflected that the time allocated to discussing and choosing one of the three alternate dietary plans (if needed) was not enough. Participants also found the concept of ‘trigger foods’ confusing, and suggested that examples of such foods and relevant replacements should be included. In addition, participants suggested that they would appreciate additional time to discuss physical activity, including the identification and discussion of barriers (analogous to existing discussions around diet). Another suggestion was that, given the amount of material to be covered in the face-to-face session, it might be helpful to be given a summary of the information in advance so that they could think about this and come prepared.

Additional comments included that the introduced concepts should be talked about in everyday language – for example referring to ‘what helps people stay on track’ rather than ‘relapse prevention’ and ‘trigger foods’. In support of the planned use of the session in the context of a broader intervention, there was a preference for ongoing follow-up or prompting to continue their weight management rather than just attending a one-off session. Most participants felt that the booklet would be a useful tool to refer back to for clarification and reminder. Finally, participants suggested that the dietary plan booklets should be made available in an electronic format (e.g., online or via smart phone/tablet app).

### 3.4. Optimisation of the intervention

#### 3.4.1. Pre-session procedures

Changes to the pre-session procedures included the addition of more detailed written information about the purpose and background of the wider study, and instructions on how to complete the four-day food diary and how to use the pedometer to count daily steps. A detailed pre-session questionnaire was also developed in order to reduce the time taken to elicit weight history and weight loss methods in the intervention session, which was then used to prompt discussion. Based on feedback regarding the desire for more focus on physical activity in the intervention and the need to acknowledge common barriers to such, a volitional help sheet for physical activity (Armitage & Arden, 2010) was also included in the pre-session pack. This help sheet contained a list of barriers or ‘if’ statements (e.g., if I feel tired) on one side and a list of solutions or ‘then’ statements (e.g., then I will remind myself that physical activity will energise me) on the other side. The participant was encouraged to link the ‘if’ and ‘then’ statements that are relevant to them by drawing a line between them to generate ideas for how to overcome barriers. This task served to both normalise the experience of encountering barriers and prompt consideration of potential solutions, which were then referred to in the intervention session during the newly included goal-setting and coping planning activity for physical activity. The decision to introduce this prior to attending the face-to-face intervention session was based on the suggestion that it would be helpful to know what was going to be covered in advance so that participants could come prepared, having already thought about the issues to be discussed in relation to their personal experiences.

#### 3.4.2. Intervention content and procedures

The biggest change to the intervention content was the decision to place less focus on specific dietetic content and recommendations (Section 3 of the original intervention). Consistent with the conceptual distinction between weight loss and maintenance, whereby the main challenge of the latter is to support ongoing, active self-regulation to enable adherence to the chosen plan, rather than the initial prescription/selection of a plan that occurs in weight loss, this was replaced with brief dietary feedback in the context of a goal-setting task. The three dietary options were then discussed *only* if a participant indicated that they were not happy with their current diet and were open to following a different plan, or if their current plan did not seem sustainable in the longer term (e.g., if they had lost weight using a very low calorie food replacement diet but were at the point of needing to transition back to regular eating). Although the plans were not made available electronically for use in the session as suggested, links to similar online dietary materials were subsequently included in the text messages received by intervention participants in the broader, multi-component intervention.

Additional changes to the content included:

- Reiterating the purpose and background of the wider study at the start of the face-to-face session to present a more comprehensive rationale for linking the use of multiple self-regulation focused techniques in relation to their WLM (e.g., goal setting for behaviour and outcome, action planning, barrier identification, and coping planning), as well as allowing the opportunity for participants to specify their own preferences and needs for support in this context;
- Expanding the explanation of ‘trigger foods’ and dedicating more time to exploring potential solutions and creating coping plans to avoid/limit consumption. In addition, a list of examples of healthy alternatives/swaps was included for a range of common trigger foods (e.g., sweet and savoury snacks), and tempting situations (e.g., eating a healthy snack prior to going out for a meal with friends so that they are less tempted to overeat). In addition to practical challenges, a number of emotion- and motivation-based challenges were also included and could be balanced with the practical challenges depending on participant needs and preferences. The language and terminology used to explain the relapse prevention tasks, and trigger foods in particular, was also modified so as to sound less technical.
- The addition of specific goal-setting tasks in relation to both eating and physical activity using a combination of the SMART goal framework (Doran, 1981) and coping planning (Kwasnicka, Presseau, White, & Sniehotta, 2013). Although dietary changes had been included in the original intervention, it was decided that more focus should be placed on specific maintenance-relevant goal setting including the rationale for doing so specifically within the context of a maintenance intervention. The volitional help sheet (Armitage & Arden, 2010) that was included in the pre-session pack was also referred to here in order to aid participants in generating coping plans for personally relevant barriers to physical activity.

#### 3.4.3. Intervention materials

Changes to the materials were made in line with the changed content described above.

### 3.5. Description of the optimised intervention

After receiving the pre-session pack in the mail (including study information, food diary, pedometer, and questionnaire), the optimised face-to-face intervention session begins with the facilitator outlining the purpose and format of the intervention to the participant, and is followed by three distinct phases and a summary (see Table 1 for outline and BCTs used in each phase). In phase one, the information provided in the pre-session questionnaire is used to briefly elicit the participants’ weight loss history and methods they have used to lose weight during their most recent attempt, including their views on why this attempt has been successful when previous attempts may not have been (i.e., encouraging them to think about mechanisms) so that subsequent tasks can be tailored to their specific support needs. The focus on past success to boost self-efficacy is retained. Participants are then asked about their current intentions regarding their weight and prompted to set a weight-related goal using the same traffic light system (i.e., regain thresholds or ‘yellow’ and ‘red’ zones; Wing et al., 2006) as in the original intervention, and the rationale for frequent self-monitoring of weight is provided.

The focus of phase two is on current diet and eating behaviour. The two relapse prevention/coping planning activities from the initial version of the intervention (trigger foods and tempting situations) are retained here, with some modifications as outlined previously. Within these tasks, participants have the opportunity to incorporate their specific needs and wants for support (e.g., practical, emotional, and social). Additionally, the rationale for goal setting using a SMART goal framework (specific, measureable, achievable, relevant, time-limited; Doran, 1981) is given to participants and they are guided through setting two maintenance-related eating goals, including specifying how, when, and where they will achieve the goal, who could help them, and anticipating potential barriers and linking these to solutions to overcome them (coping planning; Kwasnicka et al., 2013). The content of these coping plans can be related to either of the previous goals or based on any inadequacies identified in the food diary analysis. If a participant does, however, indicate that they are not satisfied with their current diet plan or it does not seem sustainable in the transition from active weight loss to WLM, they are offered a booklet on one of three alternatives: a Mediterranean diet (Esposito et al., 2011), calorie-controlled diet (Finer, 2001), and Change4Life (Department of Health, 2009), although any evidence-based plan could be suggested.

Phase three focuses on physical activity. First, a review of the participant’s current level of physical activity is undertaken (based on pedometer readings and any additional activity recorded in the study diary), alongside a discussion about the ways in which they may like to become more active and any barriers to achieving this. Current activity is then compared to the national guidelines for physical activity in adults (75 minutes of vigorous or 150 minutes of moderate activity per week, equivalent to 10,000 steps per day), and the benefits of being physically active for both health and WLM in particular are discussed. Any individual circumstances of the participant (e.g., medical risk, injuries, etc.) that may limit activity are discussed. Following the same format as the goal-setting activity for eating behaviour, participants are then prompted to set a SMART goal for increasing their physical activity, usually in the form of a minimum step count per day. Reference is made here to the volitional help sheet included in the pre-session pack in order to aid participants to generate solutions to their personally identified barriers to physical activity. The face-to-face intervention session is concluded with the facilitator summarising the participant’s goals for weight maintenance (weight zones), eating, and physical activity plans. When used in the context of the broader, 12-month, multi-component intervention, an introduction to the remainder of the intervention procedures is also included.

Consequent to the shift in focus away from dietetic information and in favour of greater focus on self-regulatory techniques, the optimised session described above could be delivered by a range of allied health professionals including but not limited to psychologists or dietitians, who have been trained using the session manual (see Supplementary File 2). The flexible nature of the intervention also means that the setting and location in which it is delivered can vary.

## 4. Discussion

The aim of this study was twofold: first, to test the acceptability and feasibility of a novel face-to-face intervention session, which would subsequently form part of a 12-month, multi-component intervention, to support the transition from successful weight loss to WLM for people who are or have been overweight/obese and have lost at least 5% of their body weight; and second, to optimise the intervention session in line with participant feedback and expert discussion for use in both the wider trial and potentially as a standalone tool. Both the initial and the optimised version of the intervention draw on strategies from self-regulation theory (Kwasnicka et al., 2016; Teixeira et al., 2015) and relapse prevention theory (Latner et al., 2013; Marlatt & Gordon, 1985), and recognise the importance of self-efficacy (Bandura, 1977) specifically for maintaining behavioural changes (Teixeira et al., 2015), which currently represent the best available evidence-based strategies for behaviour change in the area of WLM.

The optimised intervention session represents a tool that is applicable to varying populations of overweight and obese people, regardless of the method initially used to induce weight loss. This is an important distinction from many previous WLM studies, as most have included an active weight loss phase prior to concentrating on maintenance. Such programmes may therefore have incorporated some of the tasks of maintenance (e.g., switching from an approach- to an avoidance-based motivation, and from an energy deficit diet to a sustainable lifestyle) into the weight loss phase, such that people who have lost weight on their own are restricted in their access to important skills that may help the transition from weight loss to maintenance. By focusing explicitly on self-regulation, including goals for both the outcome (weight) and behaviours relevant to WLM (eating and physical activity), the current intervention session (and wider trial) fills this gap.

It is anticipated that the intervention session described here could also be used in the form of a standalone support tool (e.g., to be delivered following successful weight loss by any allied health professional or commercial weight loss consultant involved in the routine care of obesity) or as a supplement to existing weight loss and WLM support programmes (e.g., add to already available programmes currently conducted primarily online or which would typically reduce the level of support once a reasonable amount of weight has been lost). In this way, individuals are being supported not only to lose weight initially via the abundance of already available programmes and information, but also provided with tailored information and strategies for the challenging phase of maintaining weight loss. The intervention session was found to be highly acceptable to participants and feasible to deliver, and most felt that participation over a longer period of follow-up, such as that proposed for the main trial, would be beneficial.

### 4.1. Study limitations and strengths

This study had several limitations that should be considered when interpreting its findings. First, while the intervention session described here is based on best-practice guidelines and available theoretical, maintenance-relevant evidence for effectiveness, the optimised version has yet to be tested for either acceptability or effectiveness in assisting people who have successfully lost weight to avoid regain. Given that weight regain following successful weight loss usually occurs in the first year, for a maintenance intervention to be deemed successful, demonstration of results needs to occur longitudinally. Research is therefore needed to test whether involvement in such an intervention, either as a standalone tool, as a supplement to existing programmes, or as one component of a multi-component intervention (Evans et al., 2015) does have an impact on objectively measured weight and weight-relevant behaviours, both following the intervention and in the longer term. This task is currently underway in a larger trial to assess the effectiveness of an SMS-based WLM intervention, which begins with the delivery of the described optimised face-to-face session and is followed by 12 months of SMS support prior to follow-up (Evans et al., 2015).

Second, the assessment of acceptability was based on a small number of homogenous (e.g., predominantly females) and presumably quite motivated people. Given that the purpose of this study was to assess the acceptability of an early version of the intervention session rather than conduct the planned subsequent full trial of effectiveness with internal pilot study to further assess feasibility and acceptability, the sample size was deemed acceptable. Despite this, more research is required to ensure that the optimised intervention is both acceptable and effective. Additional limitations include the reliance on real-time note taking rather than audio recording the post-session interviews, and the conduct of the interviews immediately post-intervention session, which meant that participants were not able to comment on the acceptability of the suggested techniques when used outside of the session. Given that an internal pilot and process evaluation were planned as part of the wider trial (Evans et al., 2015), the current interviews were, however, deemed fit for purpose. Another limitation was the use of self-reported weights. Based on this design choice, these findings do not provide additional evidence for the acceptability of objective weighing, although findings from other studies would suggest this is both acceptable and an effective technique for managing weight when combined with other behavioural components (Madigan et al., 2014; Madigan, Daley, Lewis, Aveyard, & Jolly, 2016). Finally, further research is needed to test whether any positive results can be generalised to varying populations of obese people regardless of initial weight loss method, as was intended in the development of this tool, or whether effectiveness differs according to predictable user characteristics or preferences. This should include assessment across samples with different socioeconomic statuses to ensure that the delivery of the intervention does not widen health inequalities that already exist in obesity.

Strengths of this study include that all aspects of the intervention development process adhered to current guidelines for the design and evaluation, and reporting of complex behavioural interventions (Craig et al., 2008; Hoffmann et al., 2014) and were based on recent comprehensive reviews of evidence and theory (Dombrowski et al., 2014a; Dombrowski, Sniehotta, Avenell, et al., 2012; Teixeira et al., 2015). In particular, the use of self-regulation, self-efficacy, and relapse prevention techniques is in line with the current state of the evidence for the most effective components of WLM interventions (Dombrowski et al., 2014a, 2014b; Latner et al., 2013; Linde et al., 2006; Shin et al., 2011; Teixeira et al., 2015; Wilson et al., 2015). Further, the iterative refinement process involved a multidisciplinary collaboration to ensure that both the dietary and physical activity content, and the psychological and behaviour change elements of the intervention were comprehensive and understandable. The incorporation of such expertise with user engagement also represents a strength, by ensuring that relevant behaviour change theory, accurate and detailed nutritional and activity information, and factors that impact participant acceptability and engagement were given equal weight in the refinement and optimisation of the session for future use.

### 4.2. Conclusions

We have described the evidence-based design, piloting, and optimisation of a novel single-session, face-to-face intervention to support the transition from successful weight loss to WLM, regardless of prior weight loss method and which a range of allied health professionals and others could deliver. Preliminary results for acceptability appear promising and the incorporation of user feedback into the optimised version suggests that the tool is likely to be acceptable when used as part of the wider trial, and to go some way towards filling the current gap in the literature – that is, to provide one form of much-needed support to people who have lost weight to avoid regain, which could in turn potentially reduce both the illness and financial burden that obesity incurs for individuals, communities, and the health care system.

## Supplementary Material

Supplemental_Data.zip
