# Supplementary material for: Supporting the transition from weight loss to maintenance: development and optimisation of a face-to-face behavioural intervention component
Source: Health Psychol Behav Med. 2017 Jan 6;5(1):66–84. doi: 10.1080/21642850.2016.1269233 (PMC5297559; doi:10.1080/21642850.2016.1269233)
Supplement: Supplemental_Data.zip [file rhpb_a_1269233_sm0215.zip › Online supplementary material 2 INTERVENTION MANUAL.docx]

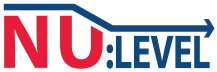


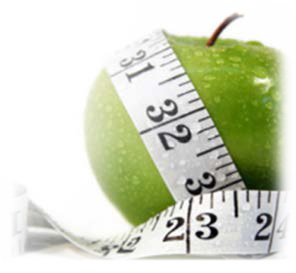


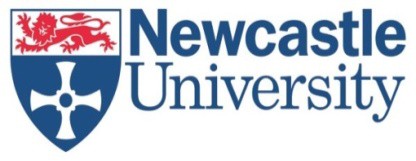


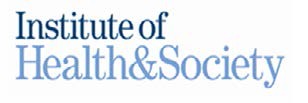


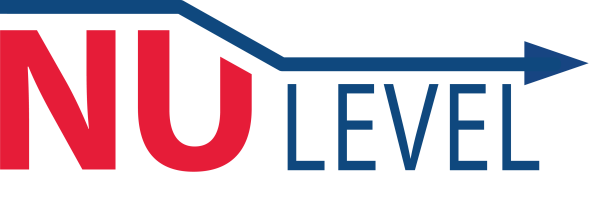


face to face intervention session: manual


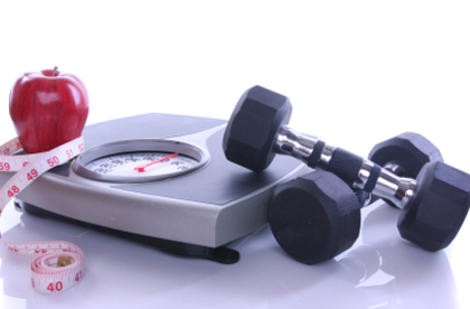


Kirby Sainsbury, Elizabeth Evans,

Falko F. Sniehotta, Vera Araujo-Soares

on behalf of the NULevel team

June 27, 2016

Newcastle University

Contents

[Overview 2](#_Toc454790177)

[Summary 2](#_Toc454790178)

[Goals of the face-to-face session 2](#_Toc454790179)

[Techniques/BCTs 3](#_Toc454790180)

[Materials: Pre-session pack (participant should bring completed pack to session) 3](#_Toc454790181)

[Materials for the session 3](#_Toc454790182)

[Before the session 4](#_Toc454790183)

[Recommended time 4](#_Toc454790184)

[Session content 4](#_Toc454790185)

[Introduction 4](#_Toc454790186)

[Agenda setting 5](#_Toc454790187)

[SECTION 1 6](#_Toc454790188)

[Introduction 6](#_Toc454790189)

[Preference for weight loss or weight loss maintenance 7](#_Toc454790190)

[Setting a weight loss maintenance goal 7](#_Toc454790191)

[Rationale for daily self-weighing 9](#_Toc454790192)

[Introduction to the interface and weight graph 10](#_Toc454790193)

[Previous weight loss success 12](#_Toc454790194)

[Questions 13](#_Toc454790195)

[SECTION 2 13](#_Toc454790196)

[Introduction 13](#_Toc454790197)

[Relapse prevention 14](#_Toc454790198)

[Setting food/eating goals 16](#_Toc454790199)

[Rationale for self-monitoring of eating and introduction to the interface 18](#_Toc454790200)

[Questions 20](#_Toc454790201)

[SECTION 3 20](#_Toc454790202)

[Introduction 20](#_Toc454790203)

[Setting an activity goal 21](#_Toc454790204)

[Linking situations with solutions (volitional help sheet for physical activity) 22](#_Toc454790205)

[Rationale for self-monitoring of activity and introduction to the interface 23](#_Toc454790206)

[CONCLUSION 25](#_Toc454790207)

# Overview

## Summary

A lot of people try and lose weight using a wide range of different methods, and many are successful. Weight loss is certainly not easy, but there are many available programmes which can lead to successful weight loss, if followed consistently. The main problem after weight loss is that most people go on to regain the weight they had previously lost, and there are no services available at the moment to help people avoid this pattern of losing weight followed by regain.

The purpose of the NULevel study is to help people to avoid regaining weight after they have successfully lost it, regardless of how they achieved their weight loss. We aim to do this by helping people to use behaviour change strategies which could support any chosen weight management plan.

This manual summarises the goals of the face-to-face intervention session, and includes information on how to deliver the session and the materials needed to do so. Throughout the manual, the blue text gives examples of the type of wording that should be used to communicate with the participant. The text in black indicates issues that need to be considered when delivering the session, as well as key ideas that should be emphasised but which can be tailored to the individual participant.

## Goals of the face-to-face session

1. Introduce participants to the content, format, and procedures of the 12-month intervention that will follow;
2. Provide the rationale for the use of the specific techniques that will be encouraged throughout the 12-month intervention (i.e., self-regulation including goal setting, action planning, coping planning, and self-monitoring);
3. To gather information from the participant about their existing goals and help them to set new goals and plans, including identifying barriers to achieving these goals and planning to overcome them, in order to personalise the intervention content and feedback.

## Techniques/BCTs

- Goal setting – outcome (weight)
- Prompt self-monitoring of behavioural outcome (weight)
- Goal setting – behaviour (eating and physical activity)
- Action planning
- Barrier identification
- Problem solving
- Coping planning/relapse prevention
- Prompt self-monitoring of behaviour (eating and physical activity)
- Provide feedback on performance
- Prompt review of behavioural goals
- Focus on past success to boost self-efficacy
- Provide information on consequences of behaviour in general
- Provide information on consequences of behaviour to the individual
- Provide instruction on how to perform the behaviour
- Provide information on where and when to perform the behaviour
- Plan social support or social change

## Materials: Pre-session pack (participant should bring completed pack to session)

- Pre-session questionnaire
- 4-day food diary, including step counts for 4 days

## Materials for the session

- Session booklet
- Screenshots of intervention interface
- Dietary booklets (calorie-controlled diet, Mediterranean diet, Change4Life or similar)
- Voice recorder

## Before the session

- Know how to introduce yourself to the participant, including your background and role in the study;
- Ensure you are familiar with the purpose of the session and how it fits in the context of the wider intervention;
- Ensure you are familiar with the specific tasks and techniques used in the session;
- Ensure you are familiar with recommendations for physical activity levels and basic dietary guidelines;
- Prepare the necessary session materials

## Recommended time

- 60-75 minutes

# Session content

## Introduction

(Approximate length: 5 minutes)

- Welcome the participant and thank them for their attendance.
- Explain your role in the study and your professional background.
- Reiterate the purpose of the study, as already explained at the baseline assessment appointment:
  - “Many people are able to lose weight using a wide range of different methods – while that’s not to say that weight loss is easy, there are many available programmes to support weight loss, most of which can help people to lose weight if they stick to them. Unfortunately, we also know that after successful weight loss, most people put the weight back on, and maintaining weight loss can be even more challenging that losing it. The purpose of the NULevel study is to help people to avoid regaining the weight they have already lost. We aim to do this by demonstrating and encouraging the use of a range of behaviour change strategies, which can be adapted and used to support any chosen weight management plan.”
- Remind the participant that only those in the intervention arm receive this session, and the text messages that will follow for the next 12-months.
- Explain the specific purpose of the face-to-face support session:
  - “The purpose of the session is to:
    1. Introduce you to the content, format, and procedures of the 12-month intervention that will follow;
    2. Explain to you why we suggest using the specific techniques that will be encouraged throughout the intervention, and which we hope you will find helpful and therefore continue using once your participation has ended; and
    3. Gather information from you so that we can make sure the intervention is meaningful to what you want to achieve, as well as complimenting the support you already have in place”
- Verbally confirm that the participant consents to take part in the face-to-face session, and is aware that they can stop at any time without giving a reason.
- Check whether the participant has any concerns or prior expectations for the session that they would like to discuss.

## Agenda setting

(Approximate length: 2 minutes)

- Explain that the support session is likely to last around an hour
- Explain the structure of the support session – the session is broken into three main sections. An introduction to the intervention website and how to use it for the various tasks will be integrated throughout. Also let the participant know that you will be writing down many of the details and plans that you talk about in the session, and will give them a copy to take away with them at the end of the session – this will serve as a reminder of what was discussed and the goals and plans they have set for their weight loss maintenance.

Section 1: Weight loss history and setting a weight goal

- Setting a weight loss maintenance goal using the traffic light system to indicate the need for more support;
- Discuss how recent weight loss was achieved, and current level of confidence for maintaining their loss

Section 2: Current diet and eating behaviour

- Review the participant’s 4-day food diary;
- Discuss a new WLM strategy (if they wish, or if it is identified that their current plan is not sustainable);
- Discuss common triggering situations and foods, and generate plans for how to deal with these in order to avoid relapse to unhealthy dietary habits;
- Identify two food-related goals and develop action plans and coping plans for each

Section 3: Physical activity

- Review the participant’s current level of physical activity, with reference to the physical activity recommendations (10,000 steps per day or 12,500 if the participant’s goal is to lose weight)
- Set a step-count goal
- Plan for physical activity setbacks

## SECTION 1: Weight loss history and setting a weight goal

(Approximate length: 15 minutes)

### Introduction

- Check that the participant received the pre-session pack in the mail, and that they have completed and brought the questionnaire and food/activity diary with them to the session
- If so, thank them for taking the time to do so
- If not, reassure them that this is okay – the purpose of the pre-session questionnaire was to get them thinking about the sorts of areas we will be covering in the session, but we will review these anyway so it is not essential (it is ideal/preferable if they do have it but we do not want to come across as punitive)
- Briefly read the relevant sections of the pre-session questionnaire (weight loss history, including methods and recent weight loss achieved)

### Preference for weight loss or weight loss maintenance

- Congratulate the participant on their recent weight loss success and ask whether they want to lose more weight or are seeking to maintain their current body weight
- If the participant’s goal is to lose further weight, make it clear that while we are completely in support of their goals, from the point of view of the study, we are primarily interested in helping them to avoid regaining what they have already lost – if this is an experience they have had before, you can link this to emphasise the importance of WLM. Explain that if/when they do successfully lose more weight during the study, we will also help them to protect any further progress. Note: for most people there is a shift in thinking needed in order to transition from WL to WLM, and this is not necessarily something that they will have thought about previously. Explain that while the weight goals we will help them set are designed to keep *maintenance* of prior loss in mind, the programme itself is not inconsistent with weight loss.

### Setting a weight loss maintenance goal

- Explain the rationale for the use of the traffic light system, linking this to the overall purpose of the intervention (i.e., to safeguard the progress the participant has already made with their weight loss):
  - “We will use a traffic light system to help you monitor your weight and progress towards your weight goals. Because the purpose of this study is to help you maintain the weight loss that you have already achieved, the green zone reflects where you are now – we want to help you stay around your current weight. The yellow and red zones are weights above where you are now, that would indicate to you that regain is beginning and that you need to act to ensure that your weight does not continue to go up.”
- Explain the idea of a ‘range of normal fluctuation’:
  - “Because there are many factors that can affect your weight, we never expect weight to be completely stable or be exactly the same each day, even when somebody is successfully maintaining their weight. For example, the time of day can have an effect – you may expect to be heavier at the end of the day than in the morning. Other factors can include whether you have emptied your bladder and bowels before stepping on the scales; the amount of water you consumed and the amount of exercise you did the previous day; your menstrual cycle (for women); as well as your food choices and a number of other things. Instead, it is normal for weight to fluctuate by up to 4-6 pounds/2-3 kilograms over the course of a week. By setting yellow and red zones, we define limits that would indicate to you that regain beyond your normal upper limit may be happening. In contrast to small fluctuations within your own predictable and acceptable range, these larger changes therefore signal the need for further action.”
- Explain that we will be providing feedback via text message, based on the weights that get sent from their study scales and the weight zones that they set in the session:
  - “By identifying potential regain early, you will be in a good position to start using dietary and behavioural strategies to prevent this weight increase from turning into more significant regain. Regular weighing and feedback acts as an ‘early warning system.’”
- Because the goal of the WLM intervention is to prevent regain, the ‘green zone’ reflects the participant’s current weight. Record the green zone/current weight in the session booklet. Explain to the participant that if they lose further weight, we can adjust this green zone downwards to reflect continued progress.
- Prompt the participant to set a ‘yellow zone’ weight (corresponding to approximately 2.5% above their current weight):
  - “I’d like you to set your yellow zone – this should be a weight that is higher than the top of your normal fluctuation and would indicate to you that you needed do something differently to prevent your weight from increasing further. Just like actual traffic lights, yellow indicates that you need to start slowing down or get ready to stop.”
  - Encourage the participant to set the zones at least 4 pounds/2kg apart, otherwise they will potentially reach their yellow zone with minimal regain and will receive inconsistent weight feedback messages Record the yellow zone weight in the session booklet.
  - Prompt the participant to set a ‘red zone’ weight (approximately 5% above their current weight) and record this in the session booklet:
  - “Now I’d like you to set your red zone weight – this should be a weight that would *definitely* signal to you that regain was happening (‘alarm bells’) and you need to act urgently in order to prevent further regain. Again, just like real traffic lights, red means you need to stop what you’re doing right away, because continuing will not lead to a good outcome!”

### Rationale for daily self-weighing

- Explain the rationale for daily self-weighing – linking self-monitoring to the weight goals/zones they have just set:
  - “We would like you to weigh yourself each day at approximately the same time. By weighing yourself daily, you will become familiar with your normal upper and lower limits, and the factors impact your weight. This awareness allows you to detect small changes in weight that are outside of this range, and puts you in the best possible position to put strategies in place to halt and reverse any regain before it becomes a more significant relapse.”
  - If the participant is concerned about daily weighing (e.g., because they have been used to weighing weekly, because they experience distress if they see an increase, or tend to become obsessive about small changes), adjust the advice on frequency of weighing to their preferences and needs – the same outcome can be achieved using variations on daily weighing:
    - “As with any advice, not everybody will find this helpful, and we would not want to insist or suggest that you do something which turns out to be counter-productive to your weight management or other factors that influence your success – for example, your mood. How would you feel about weighing yourself [e.g.] once or twice a week?”

### Introduction to the interface and weight graph

- Show the participant an example of what their weight graph will look like when they log in to the intervention interface, and explain how weight feedback will be presented to them:
  - “If you remain within your green zone (Figure 1), the programme is designed to be relatively ‘light touch’, which means you will receive some encouragement to continue managing your weight as you have been. If three consecutive weights in the yellow or red zone are recorded (Figure 2), you will receive a message letting you know that regain appears to be happening and asking what you would like to do about it – we ask that you text us back your preference: either maintain your new, higher weight (there may be reasons for this and depending on what else is going on in your life, you may just want to focus on avoiding more regain for now, rather than losing weight); or restart weight loss to return to your green zone. At this point, the intensity of the programme will increase to match your goal to lose the regained weight – this will involve receiving more frequent messages and support.
  - If you have been consistently below your green zone (Figure 3), you may be asked to change your zones down to safeguard your new weight loss. The reason we encourage you to move your zones down is that we do not want to wait until you have regained a lot of weight before our ‘alarm system’ is activated. By moving them down you can catch any regain early enough to do something about it. This is also why we encourage you to weigh yourself frequently – the more you weigh yourself, the more precise the graph will be, and the quicker we’ll be able to pick up slight increases and help you avoid reaching your yellow and red zones.”


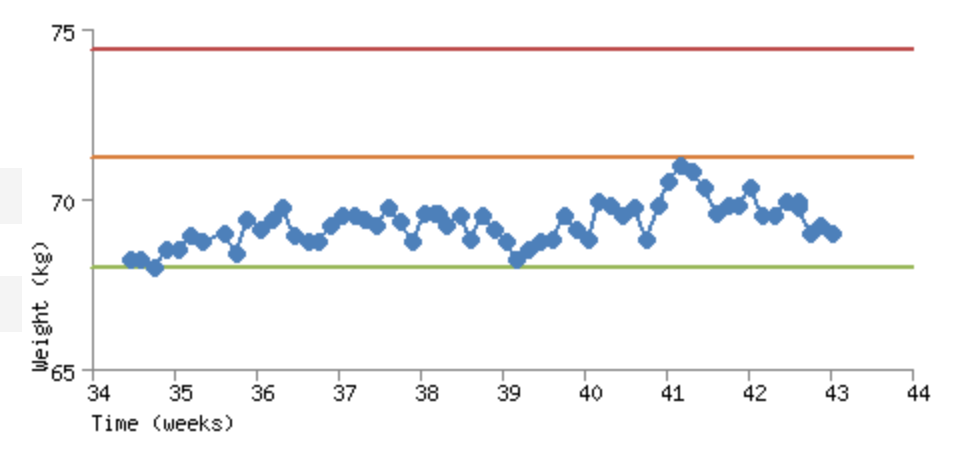


Figure 1. Maintaining in the green zone


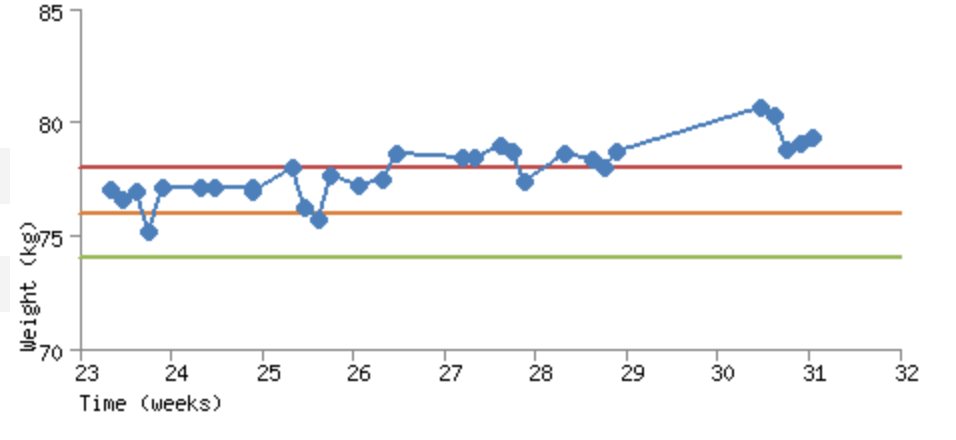


Figure 2. Regain into yellow and red zones


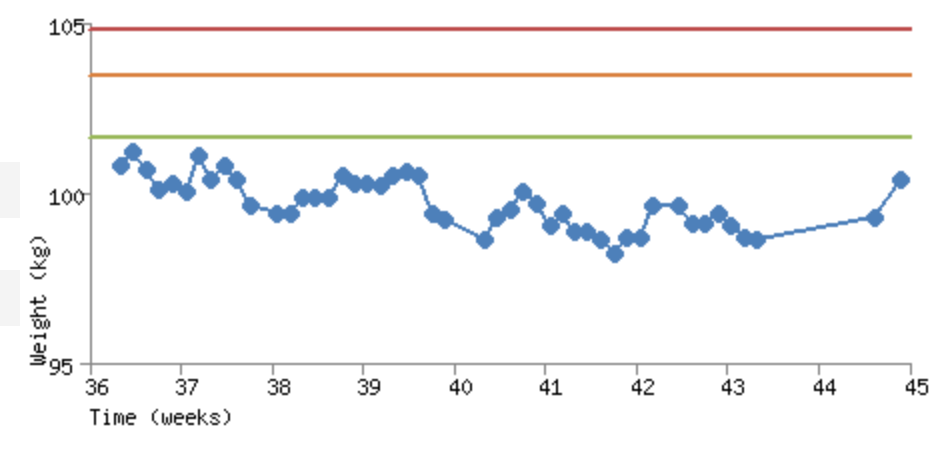


Figure 3. Consistently below the green zone

### Previous weight loss success

- Use the information in the pre-session questionnaire to prompt a brief discussion about the participant’s most recent weight loss attempt and the strategies (including their eating plan and physical activity) that they have found to be most helpful. If the participant has previously experienced failed weight loss attempt/s (either weren’t able to lose weight, or did lose weight but then regained), ask them to reflect on what was different and which led to success this time. If the participant refers to external tools/methods (e.g., Slimming World, apps such as My Fitness Pal), prompt them to identify exactly what it was about those methods that was helpful – just attending a WL programme or using an app cannot make you lose weight; how did they personally benefit from using these tools? Continue to elicit information until you have a good understanding of the ‘mechanism/s’ that led to their success.
- The main aim of this section is to *boost self-efficacy* by focusing on the past success that they have achieved – all participants in the study have already lost weight, which is a huge achievement, so there is no reason that, with the right maintenance tools and support, they cannot maintain this amazing progress. Ensure that you continue to validate their efforts and progress throughout the session (as well as throughout the following 12-months).
- Explain to the participant the reason you are interested in what has already worked for them:
  - “By identifying the strategies that have worked for you before, if you were to regain weight during the course of the programme, we would then be able to prompt you to return to what has worked for you personally in the past. Often when regain happens, all that is needed is to identify where you have slipped from your previous plan, and to make small changes again, rather than needing to completely overhaul your plan. Also, the NULevel study is focused on behavioural strategies that can be used in combination with any weight management plan – we do not advocate following any particular plan; rather, we would like you to keep doing what has already worked for you.”
- If the participant is not happy/satisfied with their existing plan or does not feel it is sustainable, tell them we can review and potentially change their specific plan in the next section.

### Questions

- Before moving on to section 2, ask if the participant has any questions; address as necessary.

## SECTION 2: Current diet and eating behaviour

(Approximate length: 20 minutes)

### Introduction

- Explain that when we talk about weight loss or WLM, we are generally referring to two sides of an equation (although it’s obviously not actually this simple) – the first is eating/food, and the second is physical activity. Section 2 of the session is focused on eating and food.
- Ask the participant if it is okay with them if you spend a couple of minutes looking at their food diary. Ask if the days they recorded are fairly ‘typical’ eating days for them. Explain that the sorts of things you are looking for include: whether they have an adequate fruit and vegetable intake; protein; or excessive intake of fats and sugars, as well as how much they are eating overall, whether they drink enough water, and how often they are eating foods that would typically be considered ‘sometimes foods’ (e.g., chocolate, crisps, takeaways etc.). Having said this, we do not advocate that the participants follow any particular type of diet – there are many different eating plans which can be used to lose weight and maintain a healthy weight.
- Ask the participant whether they are happy/satisfied with their current eating plan and whether they think it is one that they would be able to stick to in the longer-term (i.e., is it sustainable for WLM?) If the participant indicates that they are not happy with their current plan, offer to spend some time discussing some alternatives with them – for the purposes of the original NULevel study, the options were calorie-counting, the Mediterranean diet, and Change4Life (a government-funded NHS initiative). These options could be replaced with any other evidence-based, healthy eating plan.

NOTE: the order of the tasks within this section (coping planning and goal setting) can be adapted according to the needs of the participant and how the session is flowing – for example, if a review of the food diary leads into a conversation about dietary changes, then it makes sense to follow this with goal setting; whereas, in other cases it may feel more natural to lead with coping planning and have the option of basing the content of the later goal setting task on the foods/situations identified in the coping planning task.

### Relapse prevention

- Provide the rationale for relapse prevention:
  - “While you have already found a weight management plan that is working well for you, inevitably there will be things that get in the way of you being able to fulfil your plan 100% of the time. It is therefore important to anticipate common barriers that are likely to get in the way of any goal you want to achieve. This way, you can plan for how to overcome these barriers in advance of them occurring, so that you have a greater chance of being able to continue your weight management even if/when they happen. This should ensure that these things do not derail you and lead to you going off track. Today, we are going to focus on 2 barriers often encountered by people who are trying to manage their weight: specific foods and situations that place you at risk of overeating or getting off track.”

#### Trigger foods

- - Introduce the concept of a ‘trigger food’:
    - “The first barrier we are going to talk about is what we call ‘trigger foods’. These are foods and/or drinks (e.g., alcohol) that if we allow ourselves to have access to, we often find it difficult to limit how much of them we eat, and we may therefore find ourselves over-eating or eating more than we had planned. They trigger or place us at risk of lapsing back to unhealthy eating patterns. Examples might include chocolate, crisps, cake, sugary drinks, or alcohol. Are there any foods or drinks that would fall into this category for you?”
- Ask the participant to identify their trigger foods/drinks and record them in the session booklet. Ask them to recall what typically happens when they eat these foods – for example, they might intend to eat just a few but end up eating the whole bag; if they have them one day, then they want them again the next day; or eating/drinking a trigger food/drink then leads them to eating other foods they would typically limit. As part of this conversation, also prompt them to consider the points at which they might have an opportunity to break the pattern.
- Explain to the participant that there are several ways that we can manage trigger foods:
  - “Depending on your preference and what you will work best for you, there are several ways we can try to better manage trigger foods: one way is to put boundaries in place so that you *are* able to have only a small portion of the trigger food and not have this lead to you over-eating or falling into your usual pattern; the other way is to replace your trigger foods with healthy alternatives or ‘swaps’.”
- The preference for one or the other of these strategies may differ for different foods, or depending on the individual and how successful they think they are likely to be (e.g., some people will say they cannot put boundaries in place and therefore avoiding/replacing those foods is the better option; others will want to continue eating those foods in small portions because they recognise that total restriction can lead to later over-consumption) – adjust the plan for each person accordingly.
- Prompt the participant to consider a plan for how to manage their trigger foods – either by generating ideas for healthy food swaps, or by putting boundaries in place. Record their plan/s in the session booklet. If the participant is struggling to generate ideas, refer to the ‘examples of healthy food swaps for common trigger foods’ in the session booklet. Regarding boundaries, examples might include only buying a single packet of crisps or mini ice-creams rather than stocking up with family/multi-packs; or taking out a small portion and replacing the bag/packet before sitting down to eat, rather than having a whole bag next to you.

#### Tempting situations

- Introduce the concept of a ‘tempting situation’:
  - “The second common barrier I’d like to talk to you about is to do with particular situations. Similar to a trigger food that places you at risk of over-eating, a ‘tempting situation’ is one in which the *circumstances or environment* place you at risk of over-eating or lapsing back to previous unhealthy eating patterns. Common examples include socialising with friends; going on holidays; or in the context of drinking alcohol, which often impacts eating during and/or immediately after a drinking session, or the next day. Are there any situations that would fall into this category for you?”
- Ask the participant to identify their tempting situations and record them in the session booklet. Ask them to recall what has tended to happen when they have been in such situations in the past, and prompt them to consider the points at which they might have an opportunity to break the pattern.
- Use these points to prompt a discussion about the ways in which they may be able to do something differently in the future by using ‘if-then’ planning. If the participant is struggling to generate ideas, you can refer to the list of examples for ‘coping with tempting situations’ in the session booklet. Record their plans in the session booklet.

### Setting food/eating goals

- Introduce the idea of the goal setting task. The content of the participant’s goals can be something that they already know they would like to do, can be related to either of the coping planning tasks (if these were completed first), or, if they are not able to think of anything, the facilitator could suggest a goal based on the review of the participant’s food diary.
- It is important to place these in the context of maintenance – that is, for the most part we want the participant to keep doing what is already working for them, while also considering healthy changes that they may need to make to support the maintenance of their recent weight loss. The aim is not to generate entirely new behaviours that are inconsistent with their existing weight management plan, but instead that supplement what they are already doing, or, if needed, place more focus on an eating plan that is sustainable in the longer-term.
- Give the rationale for setting an eating goal/s:
  - “I’d now like to help you set yourself a goal related to your eating. By setting a goal, you draw your attention to an aspect of your behaviour that is not currently happening automatically. Once you’re thinking about it and have a plan for how to reach your goal, by repeatedly doing the new healthy behaviour, over time it should become part of your healthy set of behaviours, and become habitual so you don’t have to think about it as much. There are probably lots of things you now do as part of your healthy eating and weight management plan that at first required a lot of thought and effort, but which now you just do automatically, without thinking about it. Here, I’d like you to focus on an area of your eating that you are currently not happy with or that you would like to change – this could involve doing more or less of something, or thinking about particular situations in which you would like to do something differently. It may be something that is important for supporting your weight loss maintenance (or further weight loss, if this is their goal), or could be more related to health than your weight. Can you think of an area of your eating that you’d like to target?”
- Explain the rationale for the use of the SMART goal framework:
  - “We know that people are more likely to achieve their goals and implement their plans if the goals they set are SMART – that it, their goals are *specific* (they are clearly defined in terms of the exact behaviours and contexts that are involved); measureable (there is a clearly defined way to judge or measure progress); achievable (something that is within your reach – if a goal is too large or not currently achievable it may be better to break it into a series of smaller goals); relevant (is relevant or meaningful to a broader goal or value you hold), and time-bound (has a clearly defined time-scale for achievement). In contrast, most people are less likely to meet goals that are vague, unrealistic, or that have too wide a timeframe (e.g., in my life…).”
- Prompt the participant to generate their first goal, then guide them through the process of turning this into a SMART goal by considering the following questions/prompts. Record the goal in the session booklet.
  - What exactly are you going to do? What is the behaviour you would like to change?
  - What steps are involved? Do you need to break this down into a series of smaller steps or can they all be achieved right away?
  - Where will you do it? Do different steps happen in different places (e.g., prepare food at home to eat at work)?
  - When will you do it? (e.g., every day; on Monday, Wednesday, Friday; at lunch time during the week)
  - Is there anyone who can help you achieve it?
  - Check that the goal is indeed relevant to the participant and is something that they are motivated to work towards and achieve.
  - Prompt the participant to think about any potential barriers that are likely to get in the way of them achieving their goal, or that have previously got in the way when they have tried to enact similar plans. These should include practical barriers (e.g., availability, time) and internal barriers (e.g., forgetting, emotions/motivation). Record any barriers in the session booklet.
  - For each identified barrier, help the participant to consider how they might plan to overcome this barrier, so that they can be successful in meeting their goal (e.g., ensuring they have all the required ingredients in the house to make their planned healthy meals, batch cooking on the weekend if they are unlikely to have time during the week). Record their coping plans in the session booklet, using an ‘if-then’ format.
  - Repeat this process to generate a second eating/food-related goal and a specific set of plans for meeting their goal (action and coping).

### Rationale for self-monitoring of eating and introduction to the interface

- Present the rationale for self-monitoring of goal progression/achievement and explain how you would like the participant to do this using the intervention interface:
  - “Having set an eating goal/s and generated a plan/s for how to meet this goal, it is important to monitor your progress towards that goal. By reflecting on your progress, you can determine when your new behaviour has become habitual and perhaps no longer needs to be so closely monitored; this may be the point at which you decide to create a new goal to focus your attention on another aspect of your behaviour. Alternatively, if you have not been able to meet your goal, it is important to re-assess the goal – for example, was the goal realistic and achievable or are there a series of smaller goals you could break it into? Is the goal still relevant and something you are willing to work towards? Are there unanticipated barriers that are getting in the way of you meeting your goal, and do you need to re-develop your coping plan to overcome these?”
- Introduce the participant to how they will monitor their goals using the intervention web platform:
  - “Each Friday you will be sent a text message asking you to complete your study diary. The diary is not a food diary like the one you filled in before today’s session. Instead, we are interested in whether or not you have met your goals. For each eating goal, you will be asked whether you have met it ‘completely’, ‘partially’, or ‘not at all’. Once you have completed your weekly diary, you will be given feedback on your progress – this will be either be a ‘congratulations for having met your goals’ or, if you didn’t meet your goals, we will prompt you to consider whether there is anything additional that you can do to ensure that you meet them next week, or whether they need to be changed/adapted. If you have been consistently meeting your goals, we will also prompt you to change your goals to focus on something new.”
- Explain to the participant that they will be able to log in to the study website to view and change their goals and plans at any time (whether prompted by the research team or because they would like to change them for another reason). Show them the screen shots of the interface to demonstrate how to do this.
- Emphasise the idea that, over and above the content of the specific goals they set in this session, the self-regulatory strategies you have just discussed (i.e., goal setting, planning, anticipating barriers, coping planning, self-monitoring and revising goals as/when needed) are likely to be relevant and helpful regardless of which weight management plan they choose to follow:
  - “Regardless of how you choose to manage your weight now, it is likely that the things that have got in the way in the past were common to many plans you may have tried. The goal of the NULevel intervention is to demonstrate and encourage the development of skills that can be used to support *any* plan, and to overcome the common pitfalls that have contributed to you regaining weight in the past.” Note: if this is the first time that the participant has lost weight (i.e., does not have a history of multiple successful weight loss attempts followed by regain), then this advice will need to be adapted – this could be done by leaving out reference to previous attempts/regain, or by framing this common pattern in relation to what many other people have experienced.

### Questions

- If the participant did not previously indicate that they were dissatisfied with their current eating plan and the discussion about the three dietary plans did therefore not occur, you can offer the three dietary booklets to the participant to take away with them – reiterate the idea that we do not advocate that they follow a particular plan, but that all three booklets have some good tips and ideas for new recipes that they might like to try as part of their existing plan.
- Before moving onto section 3, ask the participant if they have any questions; address as needed.

## SECTION 3: Physical activity

(Approximate length: 15 minutes)

### Introduction

- In addition to food/eating, it is important to consider the role of physical activity in WLM. Explain to the participant why this is important:
  - “We know that making changes to eating is the main way in which successful weight loss occurs, whereas physical activity *alone* will not help you to lose weight. The role of physical activity is, however, really important when you are trying to keep weight off. In addition to weight-related benefits, being active also has benefits for overall health – for example, reducing the risk of heart disease and stroke, as well as having a positive impact on wellbeing – for example, it can be used as a stress management technique and to improve mood.”
- Review the participant’s current level of physical activity – what are their typical step counts (these should have been recorded in the 4-day food diary, using the study pedometer they received as part of the pre-session pack), and what do they do to achieve this (e.g., purposeful walking/running or incidental activity at work or around the house)? Do they do any other form of PA throughout the week (e.g., going to the gym, swimming, playing team sports etc.)? How active/sedentary are they throughout the day?
- Inform the participant about the National guidelines for physical activity – the NHS recommends that all adults take 10,000 steps per day, while more (>12,500) are recommended for weight loss or maintaining a healthy weight. If the participant is currently taking significantly less than 10,000 steps per day, reassure them that this is not uncommon – the average person in the UK takes between 3,000 and 4,000 steps per day, so most people are in the position of needing to be more active. Again, although their current activity level may not be ideal, we want to provide a validating rather than punitive environment.

### Setting an activity goal

- Explain to the participant that you would like for them to set a step-goal, and that, as with their eating goals, you would like them to monitor their progress towards this goal over the course of the intervention. You should emphasise that although the recommendations state 10,000 steps per day, it is not necessary for them to set this as their goal if they feel that it would be unachievable at this time – instead they are better off setting a lower goal that, while challenging and will require them to push themselves beyond their current activity level, is a goal that they will be able to achieve. Once they have been consistently achieving their goal, they will have the option of increasing it so that they are gradually working towards 10,000 steps per day. Note, some participants will opt to set their goal at 10,000 steps per day from the outset and use this as motivation to become more physically active. If 10,000 steps is higher than their current activity level, make sure to focus on barriers and planning solutions in the SMART goal/planning task.
- If the participant frequently engages in other forms of activity that would not be detected by the step counter (e.g., cycling or swimming), tell them that they can either set a lower step goal, knowing that their additional activity will mean they are doing the equivalent of a higher number of steps. Alternatively, they can convert their other activities into steps using the following conversion chart: <http://www.purdue.edu/walktothemoon/activities.html> (send this in an email or SMS to the participant after the session).
- Guide the participant to turn their step-related goal into a SMART goal and to create an action plan – specify how many steps they are aiming to take each day, where and when they will accumulate these steps (e.g., by going for a walk at lunchtime or in the evening), and whether there is anyone that will help them achieve their goal (e.g., a work colleague or family member).
- Having set the SMART goal and created an action plan (what, where, when etc.), prompt the participant to think about any potential barriers that are likely to get in the way of them achieving their step/activity goal, or that have previously got in the way when they have tried to enact similar plans. For each identified barrier, help the participant to consider how they might plan to overcome this barrier so that they can be successful in meeting their goal (e.g., ensuring that they have comfortable walking shoes with them so they can walk home from work). Record their plans in the session booklet, using an ‘if-then’ format for the coping planning section.
- If the participant would like to set another physical activity goal (e.g., go to the gym twice per week, or build up to running 5km), repeat this process using the second goal sheet in the session booklet.

### Linking situations with solutions (volitional help sheet for physical activity)

- This section can be integrated with the goal setting task if the participant is struggling to identify potential barriers to meeting their goals, or if, having identified the barriers, they are struggling to generate solutions to these.
- Refer the participant back to the volitional help sheet that was included in their pre-session pack and explain its purpose/how to use it:
  - “You will have already seen this sheet in your pre-session pack, but I just want to spend a couple of minutes explaining it. On one side, the help sheet contains a list of commonly encountered barriers or ‘if’ statements; and on the other side, it has a list of potential solutions or ‘then’ statements. While you may sometimes encounter a barrier that is very specific to you and therefore requires a highly personalised solution to be overcome, the majority of barriers to physical activity are likely to be relevant to most people – for example, the weather or feeling tired are things that many people will come up against. Obviously not all solutions will be appropriate to each of the barriers, but this list can be useful in identifying barriers and solutions that you may not have otherwise thought of. We know from research that people are more likely to successfully follow through and meet their goals if they have already anticipated likely barriers and planned solutions to overcome these if/when they occur. By linking the barriers that are personally relevant to you to suggested solutions, you will hopefully be more likely to meet your activity goals.”
- The other purpose of the help sheet in the context of the intervention is to normalise the experience of encountering barriers to physical activity – emphasise to the participant that if they have experienced any of these barriers, they are not alone, as many people encounter these barriers frequently:
  - “Rather than the experience of encountering a barrier per se, it is how you overcome these barriers that will determine whether or not you are able to successfully meet your goals.”

### Rationale for self-monitoring of activity and introduction to the interface

- - Similar to that in the eating behaviour section, present the rationale for self-monitoring of goal progression/achievement and explain how you would like the participant to do this using the intervention interface:
    - “As I mentioned before in relation to your eating goals, we will send you a text message every Friday prompting you to complete your diary. When you follow the link to the diary, you will also be asked to input your daily step count for the last 7 days – the pedometer is able to store data for up to 7 days so you only need to do this once a week. Once you have begun inputting step counts, a graph will be generated (see Figure 4), which shows you whether you are meeting your step-count goal. The line across the graph indicates where we have set your personal step goal. Even if you are not meeting your goal consistently every day, goal achievement is worked out on average across the week, so you can compensate for less active days by being more active on others and still meet your goal.”


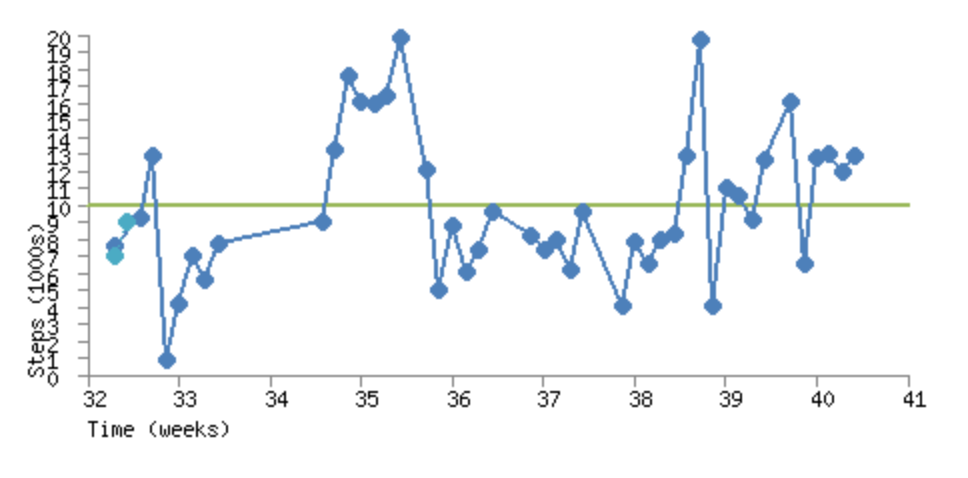


Figure 4. Daily step count and goal achievement

- Explain that, as with their eating goals, having completed their weekly diary, they will be given feedback on their progress – either congratulated on having met their goal, and after having consistently met it, prompted to increase their step goal; or if they have not met their goal, being prompted to consider whether there is anything additional they can do to ensure they meet it next week, or whether it needs to be changed/adapted.
- Explain that they will also be able to log in to the interface to view and change their goals and plans at any time (whether prompted by the research team or because they would like to change them for another reason). Show them the screen shots of the interface to demonstrate how to do this.

## CONCLUSION

(Approximate length: 10 minutes)

- - - - Explain the remainder of the weekly diary to participants:
        - “As well as reporting on your eating and activity goals, we will also be asking you to rate each of the following factors each week: your physical and emotional wellbeing and energy levels over the past week; how much of a priority your weight management is to you this week; how confident you feel and how much you feel like you know what to do to maintain your weight over the next week. Your answers to these questions will provide us with some extra context when we are sending feedback to you – for example, if you have rated your physical wellbeing and energy as much lower than usual, we will not send messages encouraging you to increase your physical activity if you didn’t meet your goal, because you were clearly unwell. It will also give us the ability to determine the factors that are related to you being more or less successful on a week-to-week basis in your weight management.”
- Explain that the intervention is designed to be relatively ‘light touch’ while they are doing well (maintaining in the green zone and meeting goals), and increases in intensity if/when regain begins to occur. It is up to the participant how much text-based contact they would like to have with the research team – in addition to responding to specific automated text messages, they can also reply at any time if they would like to ask a question or express any concerns.
- Explain that following the face-to-face session we will send them a range of text messages, including prompts to weigh themselves (initially daily and then decreasing to only if no weights are received for 4 consecutive days); prompts to complete their weekly diary; feedback on weight and weight zone changes; feedback on progress towards goals (eating, physical activity); prompts to change goals if they have been consistently met; as well as content messages focused on motivation, habit formation, managing competing priorities and stressful situations, and social support, and links to useful websites featuring recipes and other tips.
- If they would like to contact us at any time, they can reply to any of our messages and we will get back to them ASAP (messages are checked daily) – this could be if they are having technical problems, have a question, or need additional support. When they submit their weekly diary, there is also the option to tick a ‘request for contact’ box, and a member of the research team will get back to them ASAP.
- In addition, they are able to log in to the study website at any time to view their weight graph and view/change their eating and activity goals. If they want to make changes to their weight zones they will need to message us and we will make the changes for them.
- Summarise the participant’s weight, eating, and activity goals and double check that they are correct.
- Let the participant know that after the session you will type all of the information about their goals and plans into their profile on the website and let them know when their profile is ready and they can log in. Ensure the participant knows their username and password – you can write this on the front page of the study booklet so it is easily accessible.
- Before closing the session, ask whether the participant has any final questions; answer as needed.
- Thank them for their time and let them know you will be in touch via text message soon.
