# Supplementary material for: Supporting the transition from weight loss to maintenance: development and optimisation of a face-to-face behavioural intervention component
Source: Health Psychol Behav Med. 2017 Jan 6;5(1):66–84. doi: 10.1080/21642850.2016.1269233 (PMC5297559; doi:10.1080/21642850.2016.1269233)

## Slide 1
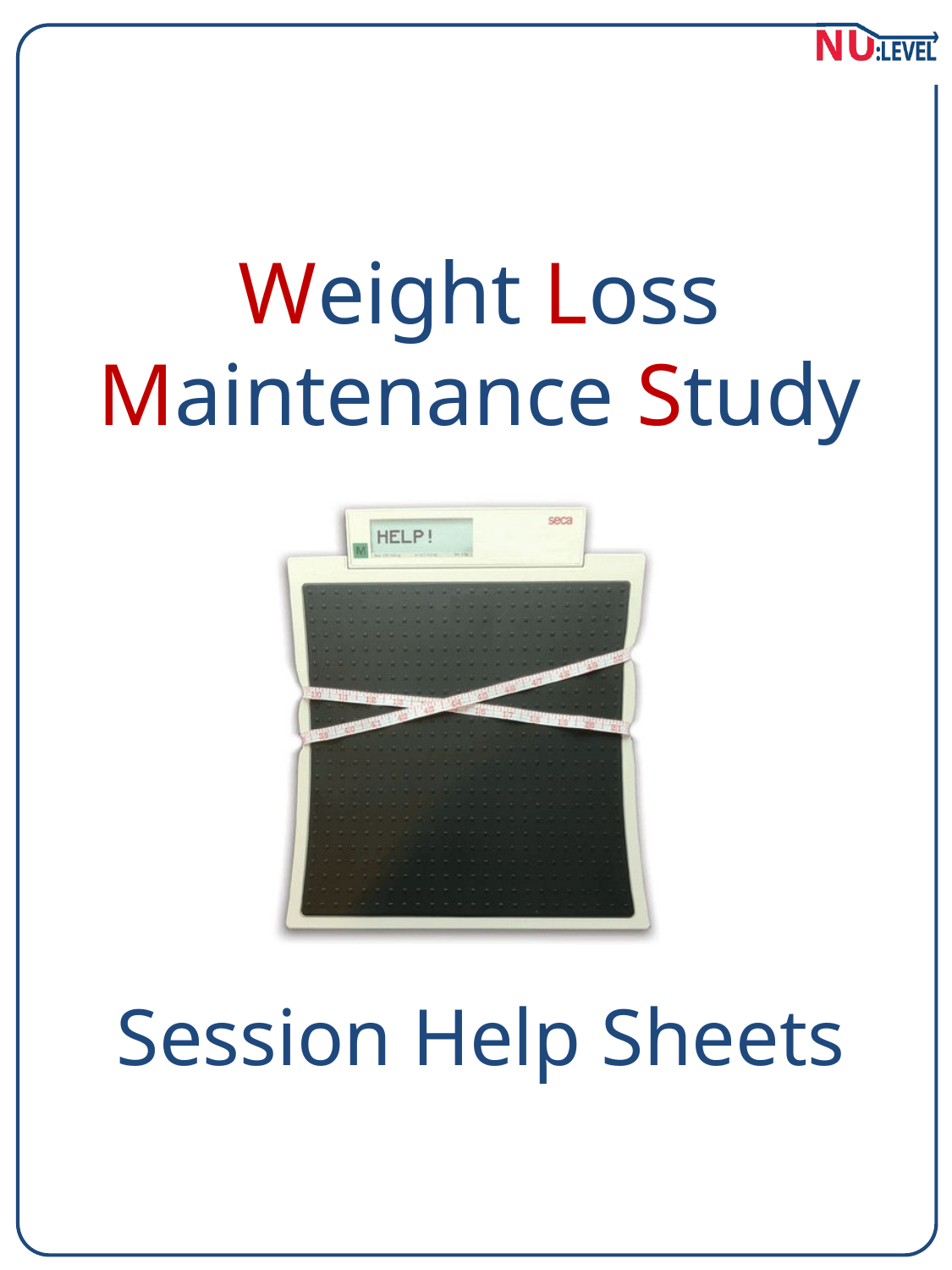

# Weight Loss Maintenance Study
Session Help Sheets

## Slide 2
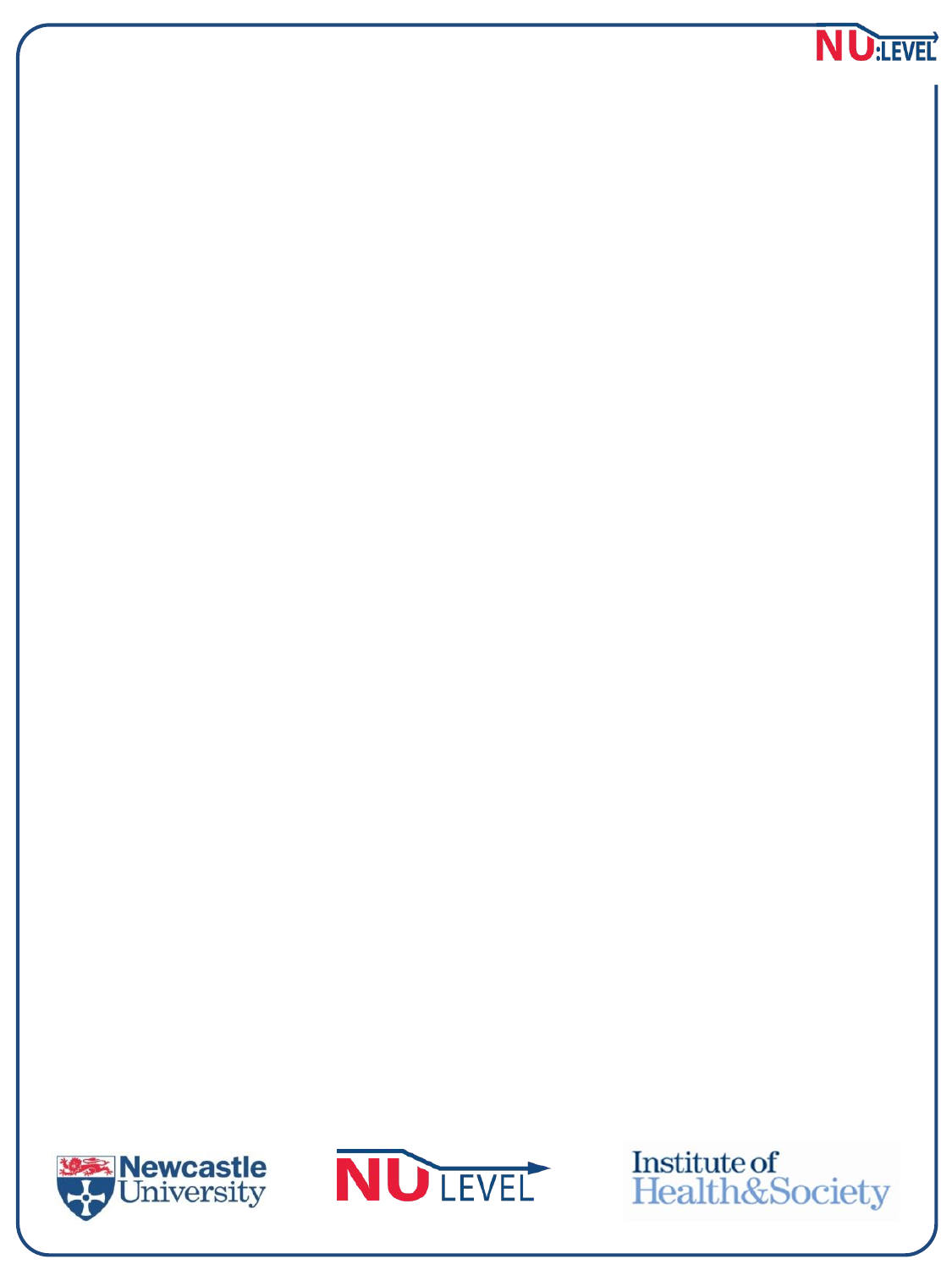

## Slide 3
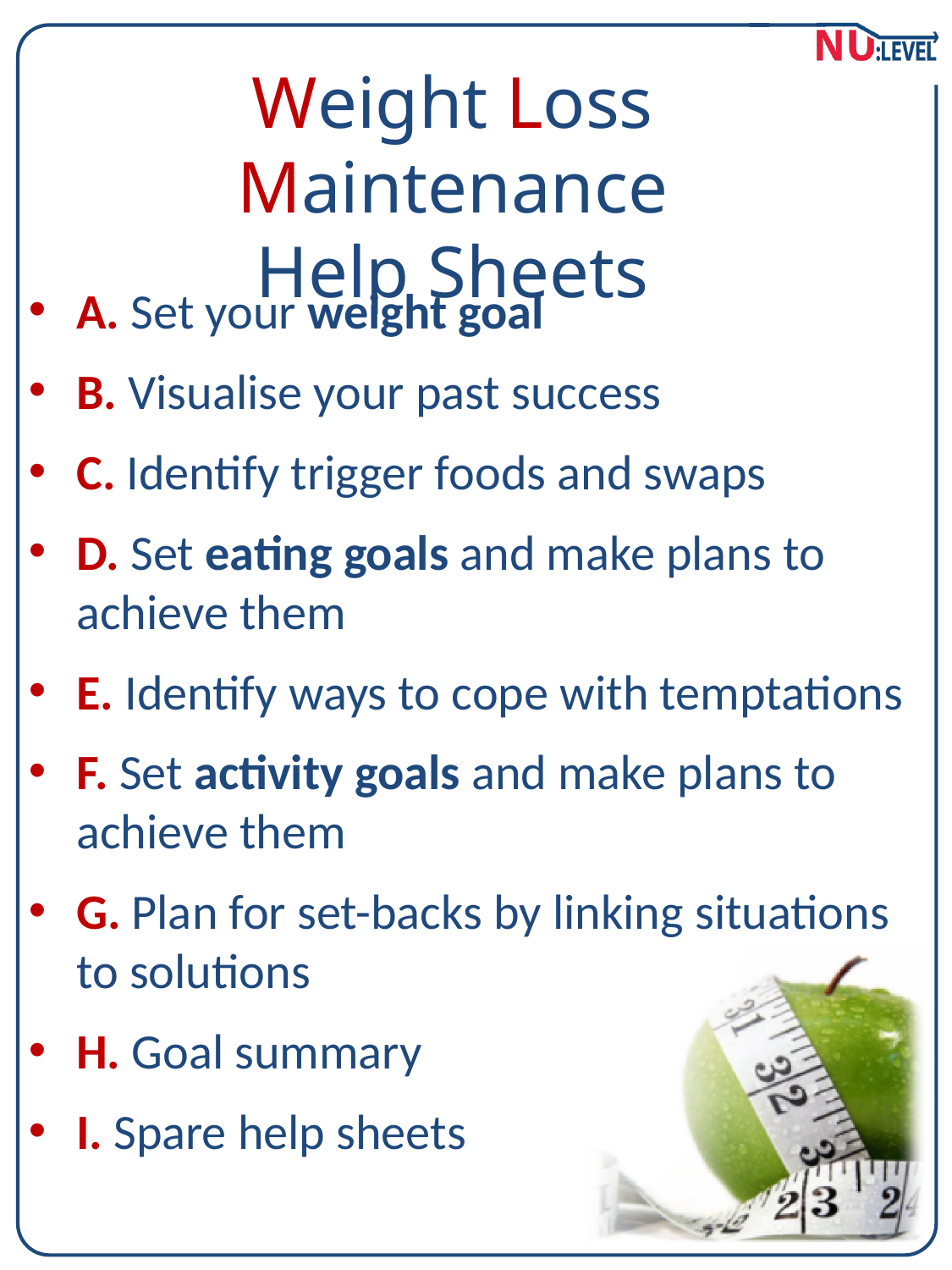

Weight Loss Maintenance
Help Sheets
A. Set your weight goal
B. Visualise your past success
C. Identify trigger foods and swaps
D. Set eating goals and make plans to achieve them
E. Identify ways to cope with temptations
F. Set activity goals and make plans to achieve them
G. Plan for set-backs by linking situations to solutions
H. Goal summary
I. Spare help sheets

## Slide 4
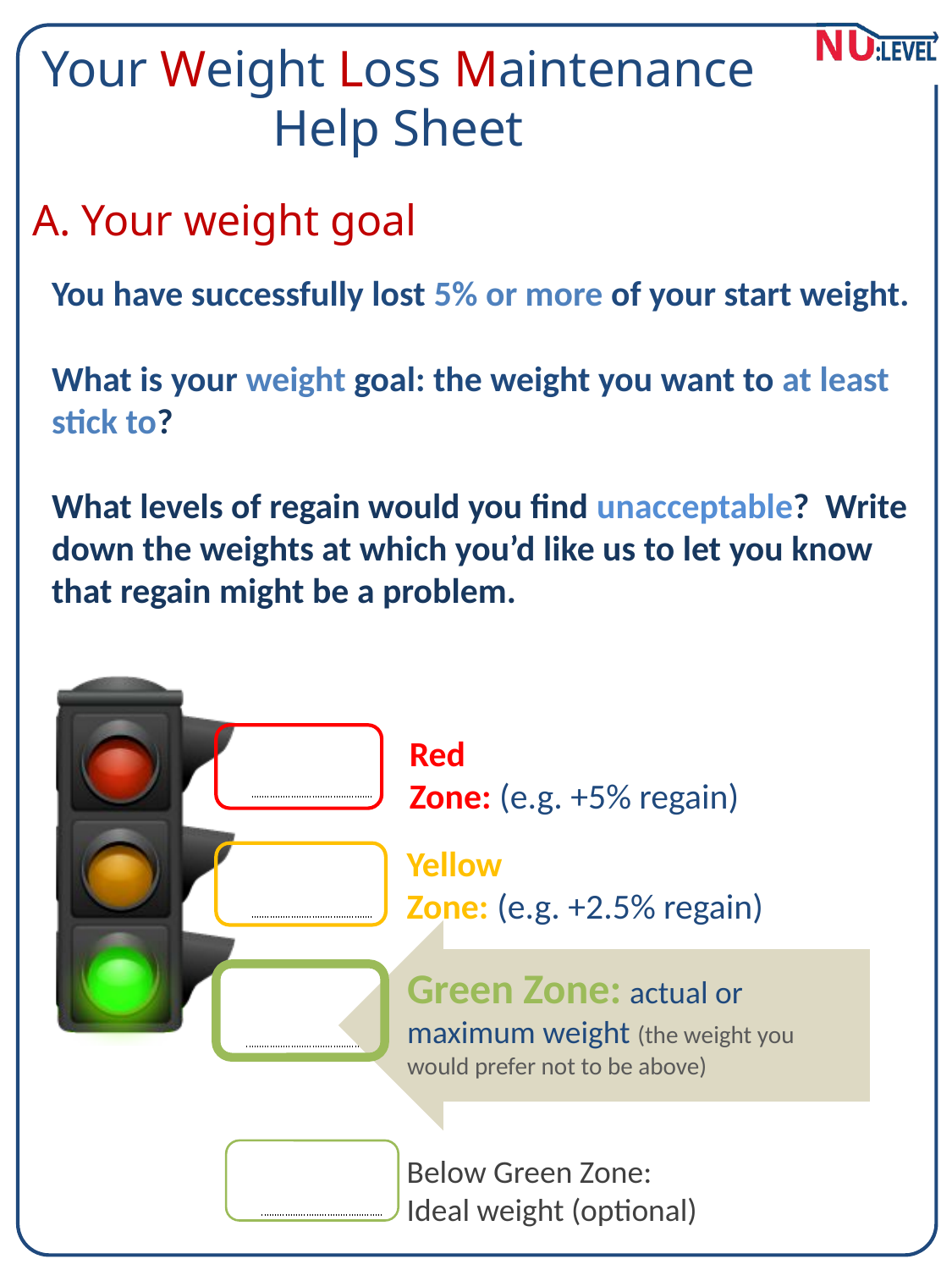

Your Weight Loss Maintenance Help Sheet
A. Your weight goal
You have successfully lost 5% or more of your start weight.
What is your weight goal: the weight you want to at least stick to?
What levels of regain would you find unacceptable? Write down the weights at which you’d like us to let you know that regain might be a problem.
Red
Zone: (e.g. +5% regain)
| |
| --- |
Yellow
Zone: (e.g. +2.5% regain)
| |
| --- |
Green Zone: actual or maximum weight (the weight you would prefer not to be above)
| |
| --- |
Below Green Zone:
Ideal weight (optional)
| |
| --- |

## Slide 5
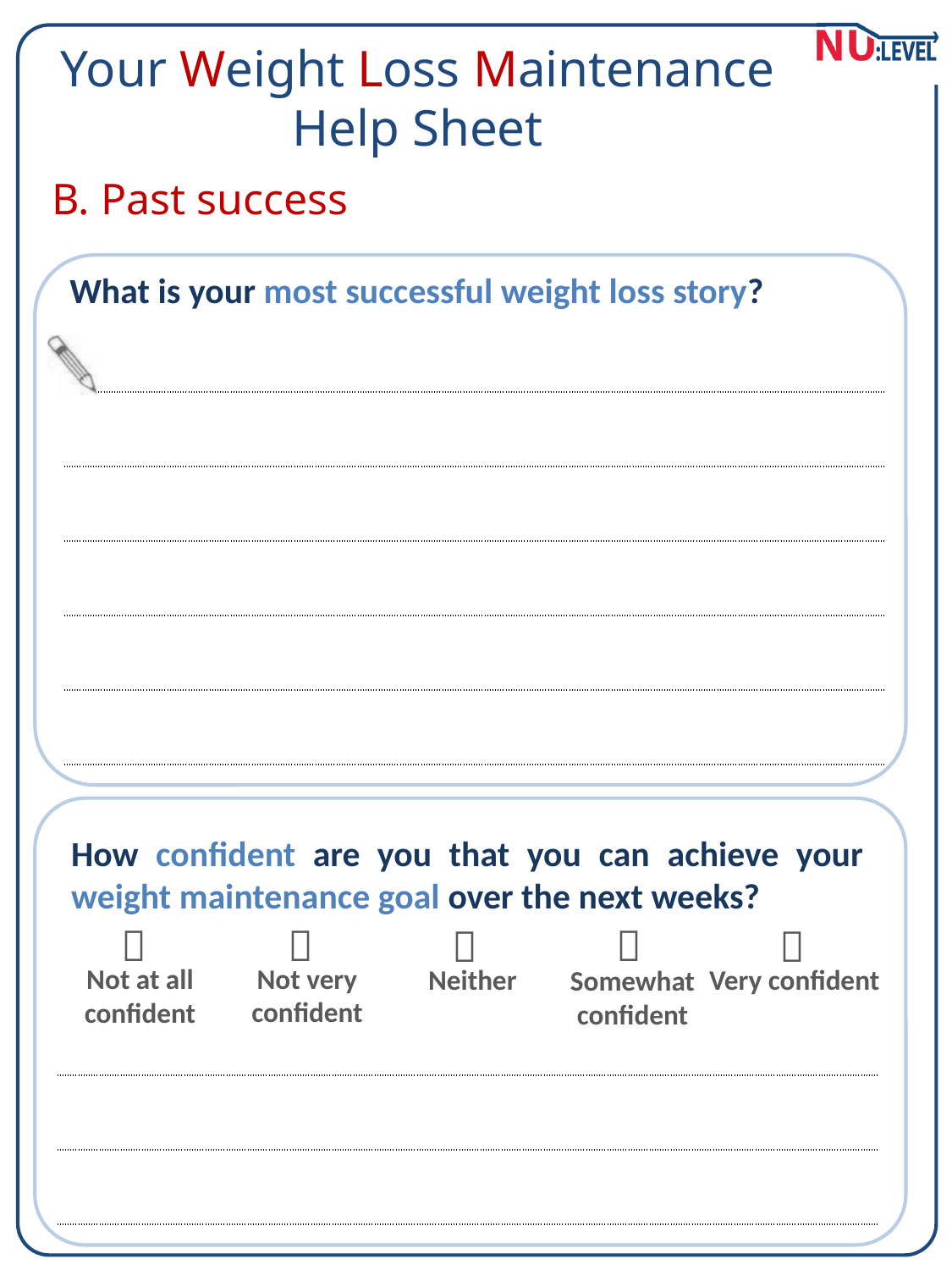

Your Weight Loss Maintenance Help Sheet
B. Past success
What is your most successful weight loss story?
| |
| --- |
| |
| |
| |
| |
| |
How confident are you that you can achieve your weight maintenance goal over the next weeks?





Not very confident
Not at all confident
Neither
Very confident
Somewhat confident
| |
| --- |
| |

## Slide 6
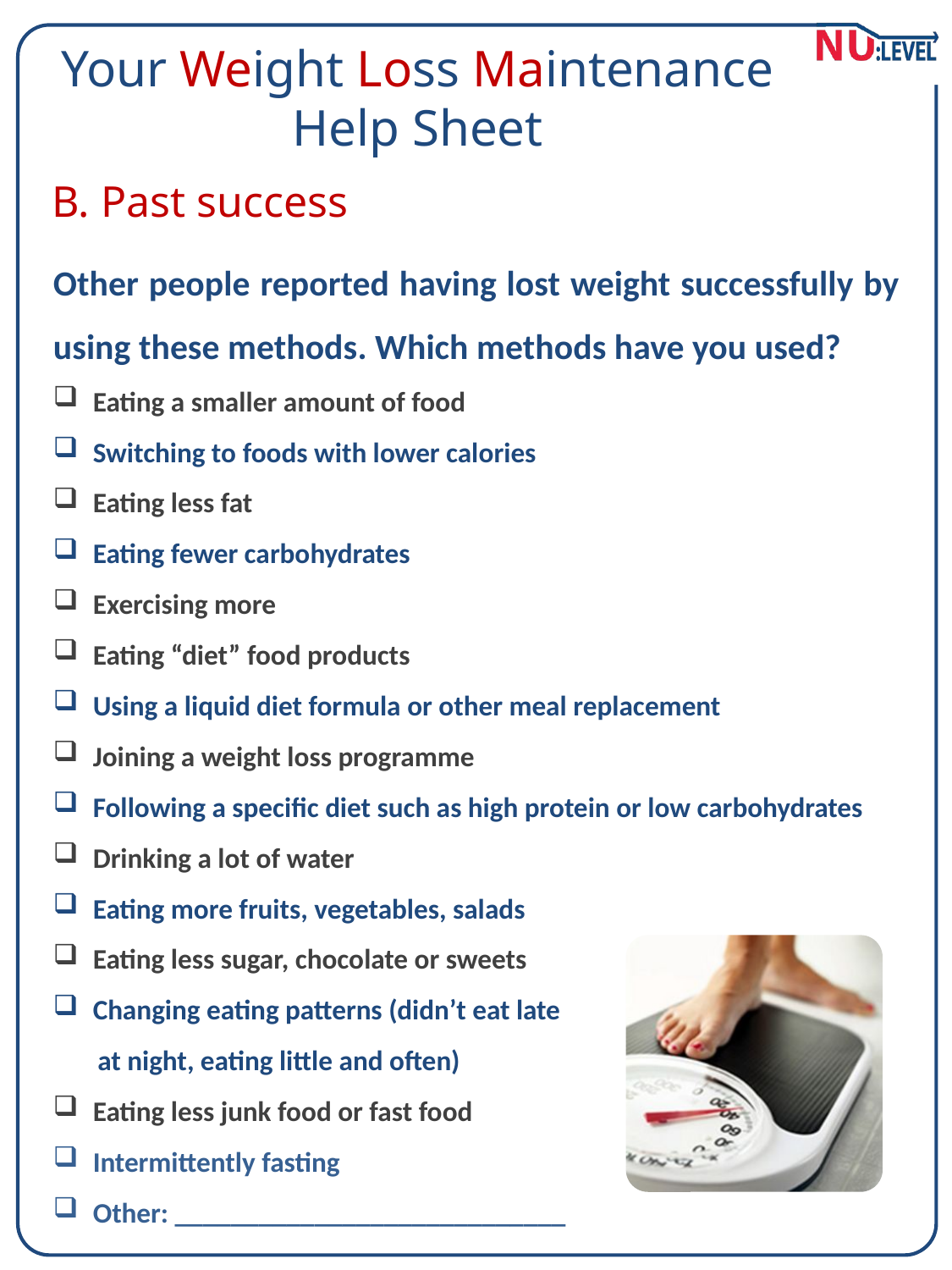

Your Weight Loss Maintenance Help Sheet
B. Past success
B. Past success
Other people reported having lost weight successfully by using these methods. Which methods have you used?
Eating a smaller amount of food
Switching to foods with lower calories
Eating less fat
Eating fewer carbohydrates
Exercising more
Eating “diet” food products
Using a liquid diet formula or other meal replacement
Joining a weight loss programme
Following a specific diet such as high protein or low carbohydrates
Drinking a lot of water
Eating more fruits, vegetables, salads
Eating less sugar, chocolate or sweets
Changing eating patterns (didn’t eat late
 at night, eating little and often)
Eating less junk food or fast food
Intermittently fasting
Other: ____________________________

## Slide 7
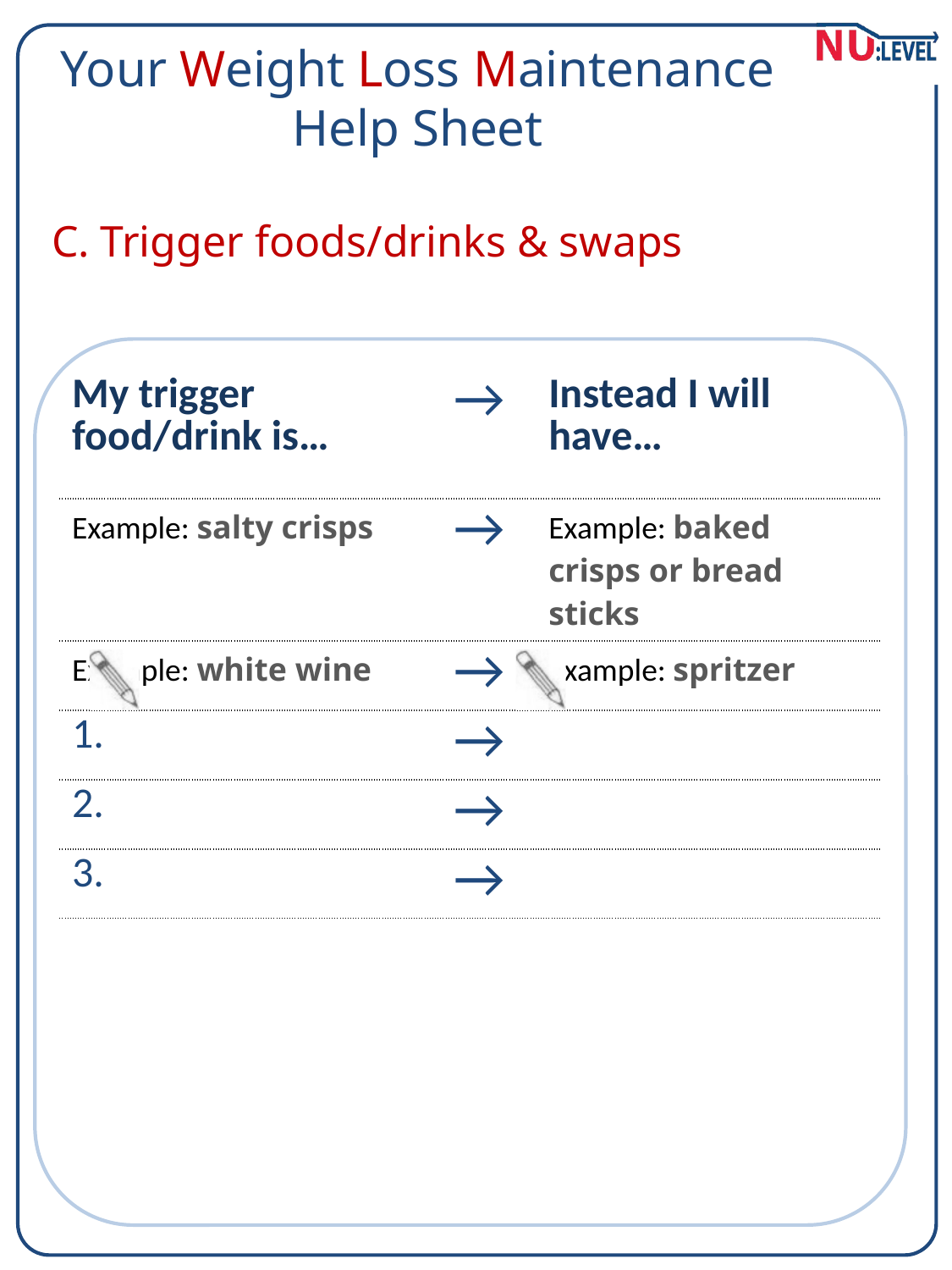

Your Weight Loss Maintenance Help Sheet
C. Trigger foods/drinks & swaps
| My trigger food/drink is… | → | Instead I will have… |
| --- | --- | --- |
| Example: salty crisps | → | Example: baked crisps or bread sticks |
| Example: white wine | → | Example: spritzer |
| 1. | → | |
| 2. | → | |
| 3. | → | |

## Slide 8
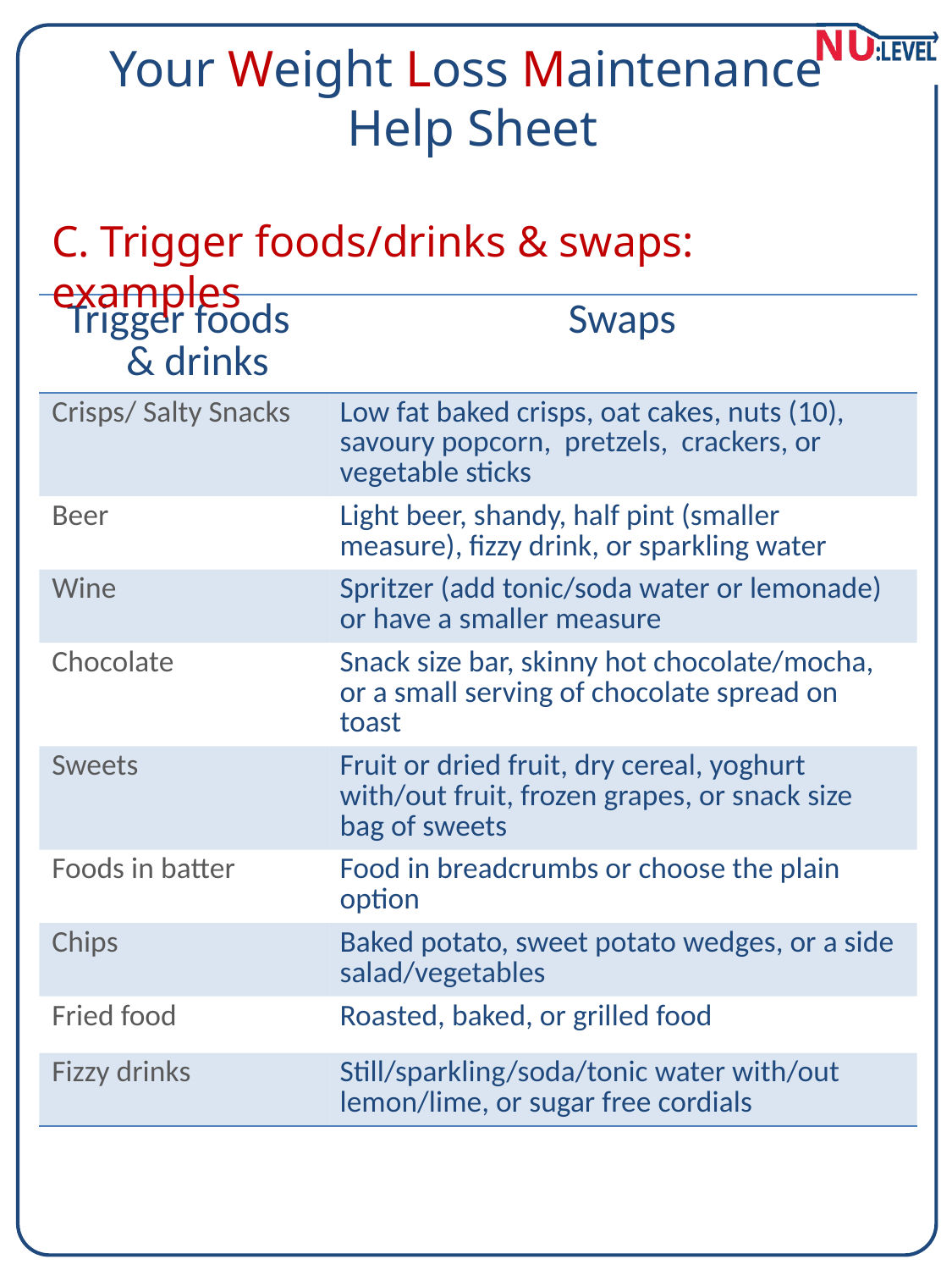

Your Weight Loss Maintenance
Help Sheet
C. Trigger foods/drinks & swaps: examples
| Trigger foods & drinks | Swaps |
| --- | --- |
| Crisps/ Salty Snacks | Low fat baked crisps, oat cakes, nuts (10), savoury popcorn, pretzels, crackers, or vegetable sticks |
| Beer | Light beer, shandy, half pint (smaller measure), fizzy drink, or sparkling water |
| Wine | Spritzer (add tonic/soda water or lemonade) or have a smaller measure |
| Chocolate | Snack size bar, skinny hot chocolate/mocha, or a small serving of chocolate spread on toast |
| Sweets | Fruit or dried fruit, dry cereal, yoghurt with/out fruit, frozen grapes, or snack size bag of sweets |
| Foods in batter | Food in breadcrumbs or choose the plain option |
| Chips | Baked potato, sweet potato wedges, or a side salad/vegetables |
| Fried food | Roasted, baked, or grilled food |
| Fizzy drinks | Still/sparkling/soda/tonic water with/out lemon/lime, or sugar free cordials |

## Slide 9
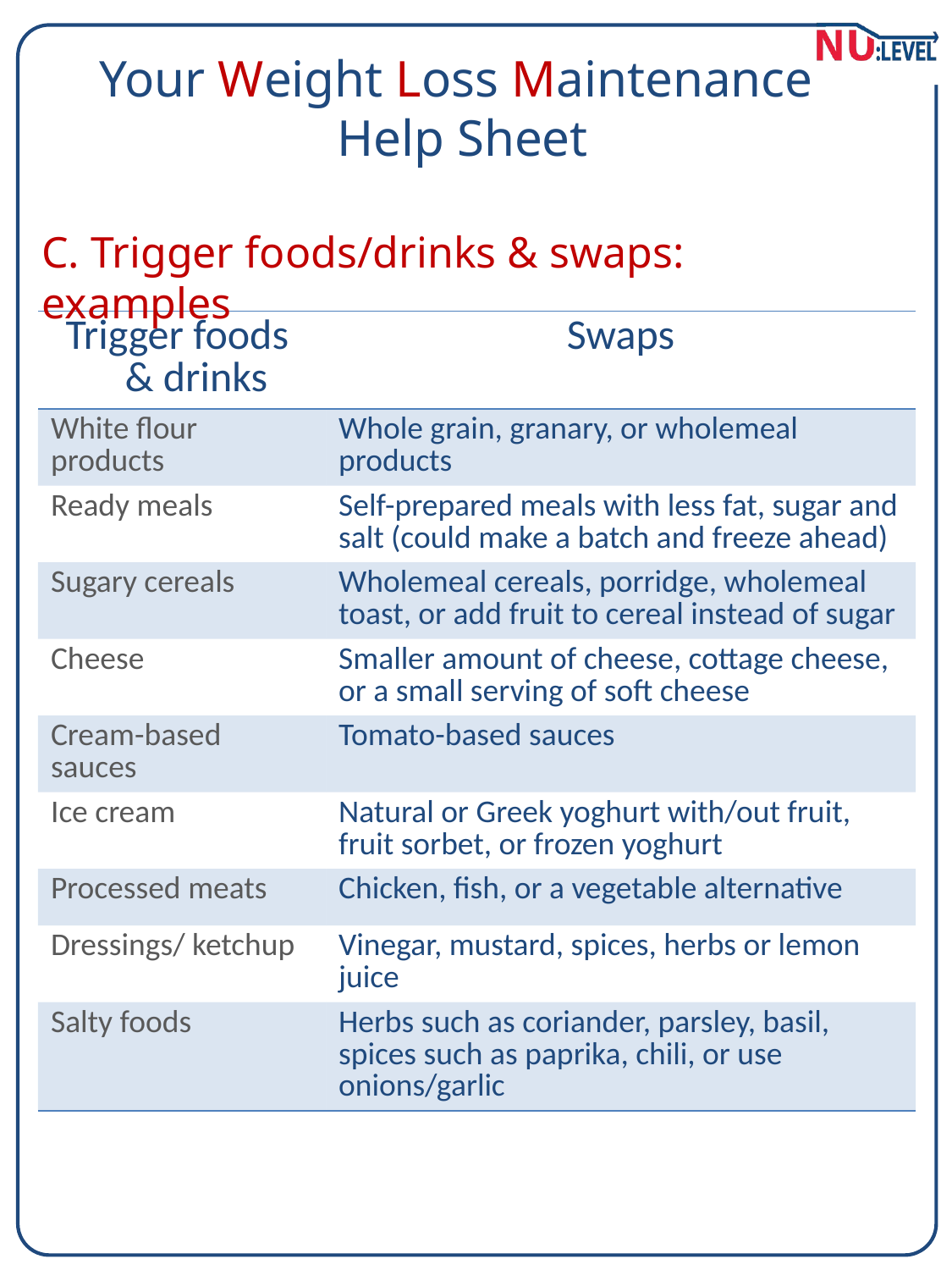

Your Weight Loss Maintenance
Help Sheet
C. Trigger foods/drinks & swaps: examples
| Trigger foods & drinks | Swaps |
| --- | --- |
| White flour products | Whole grain, granary, or wholemeal products |
| Ready meals | Self-prepared meals with less fat, sugar and salt (could make a batch and freeze ahead) |
| Sugary cereals | Wholemeal cereals, porridge, wholemeal toast, or add fruit to cereal instead of sugar |
| Cheese | Smaller amount of cheese, cottage cheese, or a small serving of soft cheese |
| Cream-based sauces | Tomato-based sauces |
| Ice cream | Natural or Greek yoghurt with/out fruit, fruit sorbet, or frozen yoghurt |
| Processed meats | Chicken, fish, or a vegetable alternative |
| Dressings/ ketchup | Vinegar, mustard, spices, herbs or lemon juice |
| Salty foods | Herbs such as coriander, parsley, basil, spices such as paprika, chili, or use onions/garlic |

## Slide 10
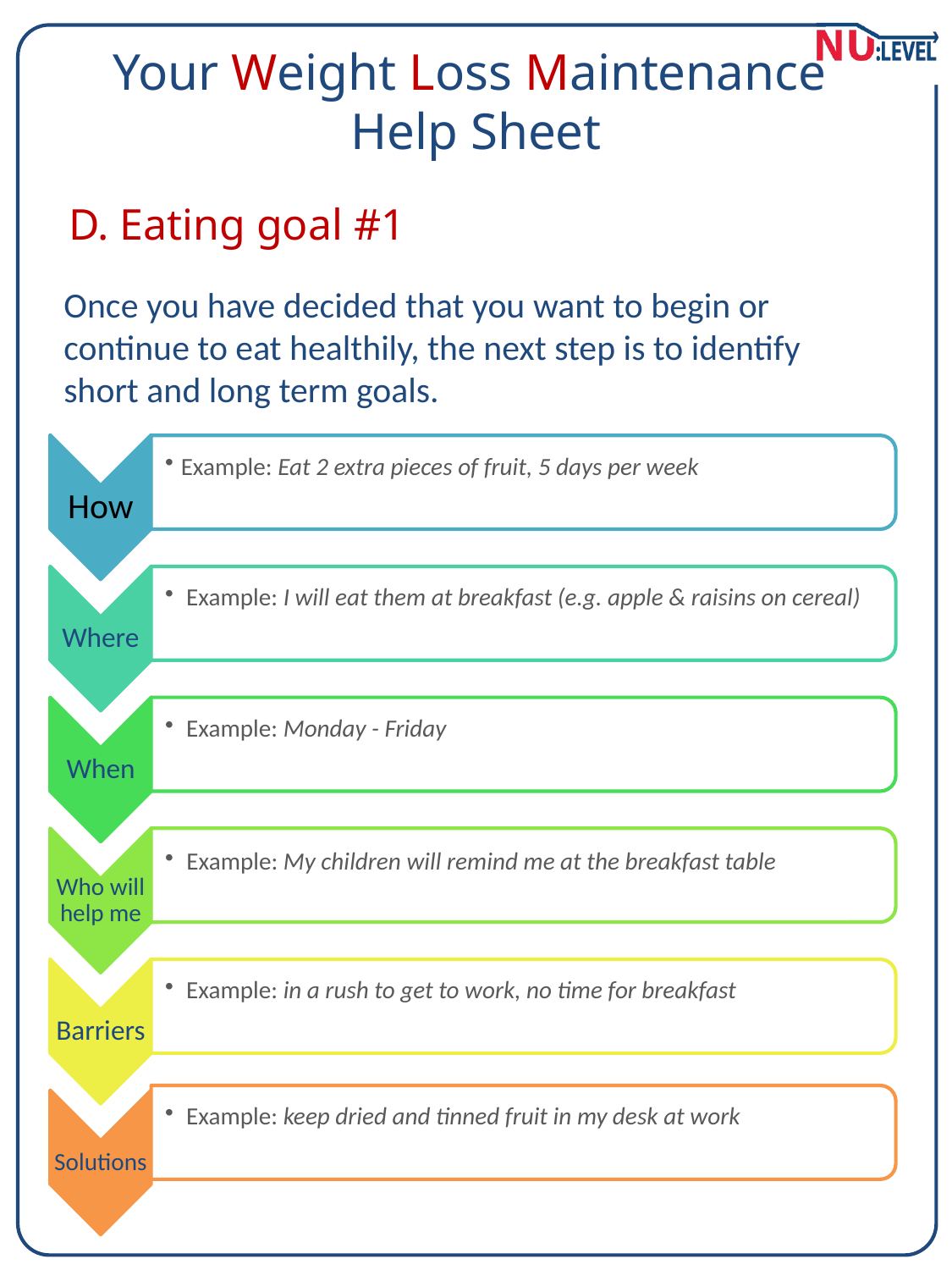

Your Weight Loss Maintenance
Help Sheet
D. Eating goal #1
Once you have decided that you want to begin or continue to eat healthily, the next step is to identify short and long term goals.

## Slide 11
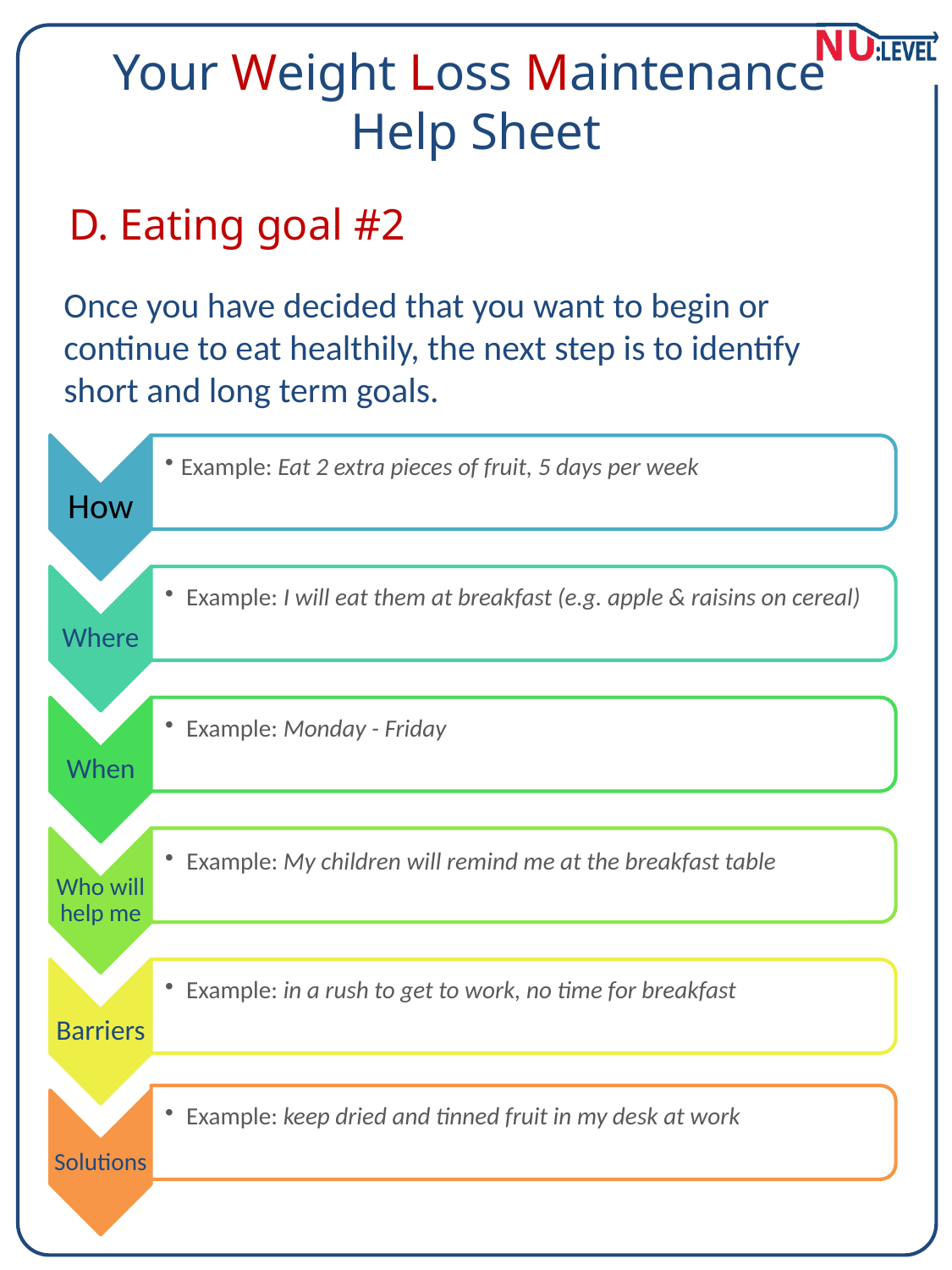

Your Weight Loss Maintenance
Help Sheet
D. Eating goal #2
Once you have decided that you want to begin or continue to eat healthily, the next step is to identify short and long term goals.

## Slide 12
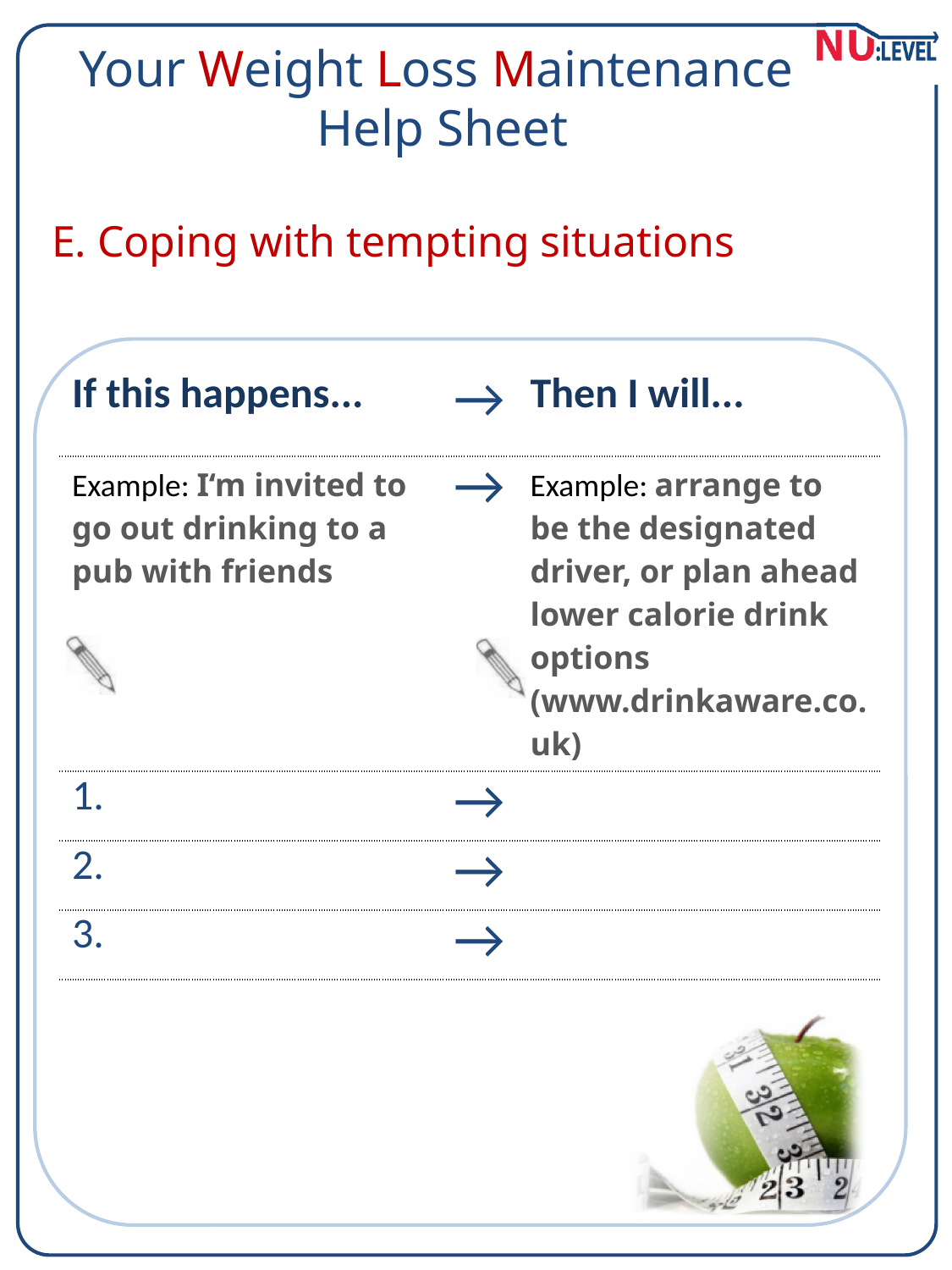

Your Weight Loss Maintenance
Help Sheet
E. Coping with tempting situations
| If this happens... | → | Then I will... |
| --- | --- | --- |
| Example: I‘m invited to go out drinking to a pub with friends | → | Example: arrange to be the designated driver, or plan ahead lower calorie drink options (www.drinkaware.co.uk) |
| 1. | → | |
| 2. | → | |
| 3. | → | |

## Slide 13
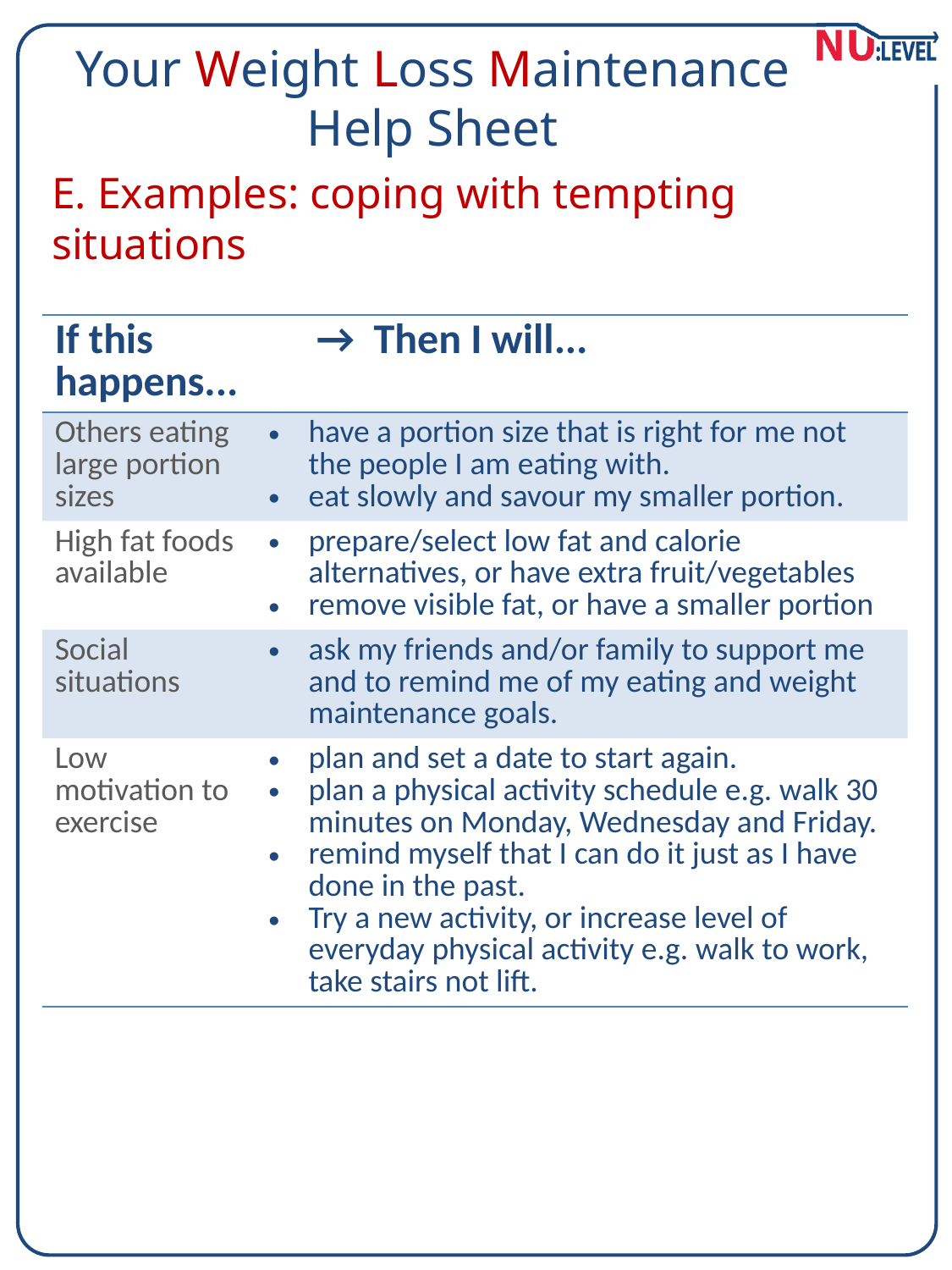

Your Weight Loss Maintenance Help Sheet
E. Examples: coping with tempting situations
| If this happens... | → Then I will... |
| --- | --- |
| Others eating large portion sizes | have a portion size that is right for me not the people I am eating with. eat slowly and savour my smaller portion. |
| High fat foods available | prepare/select low fat and calorie alternatives, or have extra fruit/vegetables remove visible fat, or have a smaller portion |
| Social situations | ask my friends and/or family to support me and to remind me of my eating and weight maintenance goals. |
| Low motivation to exercise | plan and set a date to start again. plan a physical activity schedule e.g. walk 30 minutes on Monday, Wednesday and Friday. remind myself that I can do it just as I have done in the past. Try a new activity, or increase level of everyday physical activity e.g. walk to work, take stairs not lift. |

## Slide 14
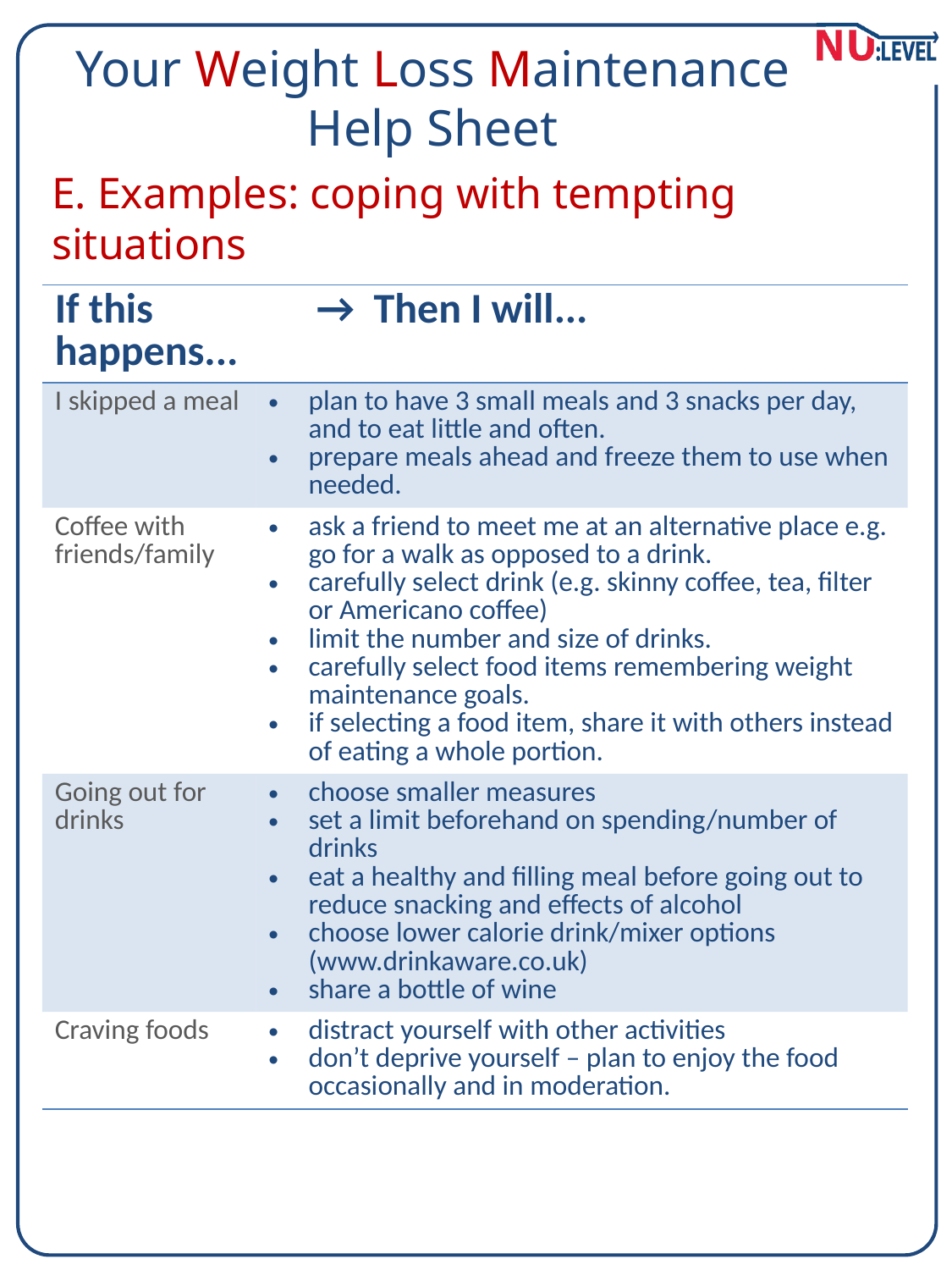

Your Weight Loss Maintenance Help Sheet
E. Examples: coping with tempting situations
| If this happens... | → Then I will... |
| --- | --- |
| I skipped a meal | plan to have 3 small meals and 3 snacks per day, and to eat little and often. prepare meals ahead and freeze them to use when needed. |
| Coffee with friends/family | ask a friend to meet me at an alternative place e.g. go for a walk as opposed to a drink. carefully select drink (e.g. skinny coffee, tea, filter or Americano coffee) limit the number and size of drinks. carefully select food items remembering weight maintenance goals. if selecting a food item, share it with others instead of eating a whole portion. |
| Going out for drinks | choose smaller measures set a limit beforehand on spending/number of drinks eat a healthy and filling meal before going out to reduce snacking and effects of alcohol choose lower calorie drink/mixer options (www.drinkaware.co.uk) share a bottle of wine |
| Craving foods | distract yourself with other activities don’t deprive yourself – plan to enjoy the food occasionally and in moderation. |

## Slide 15
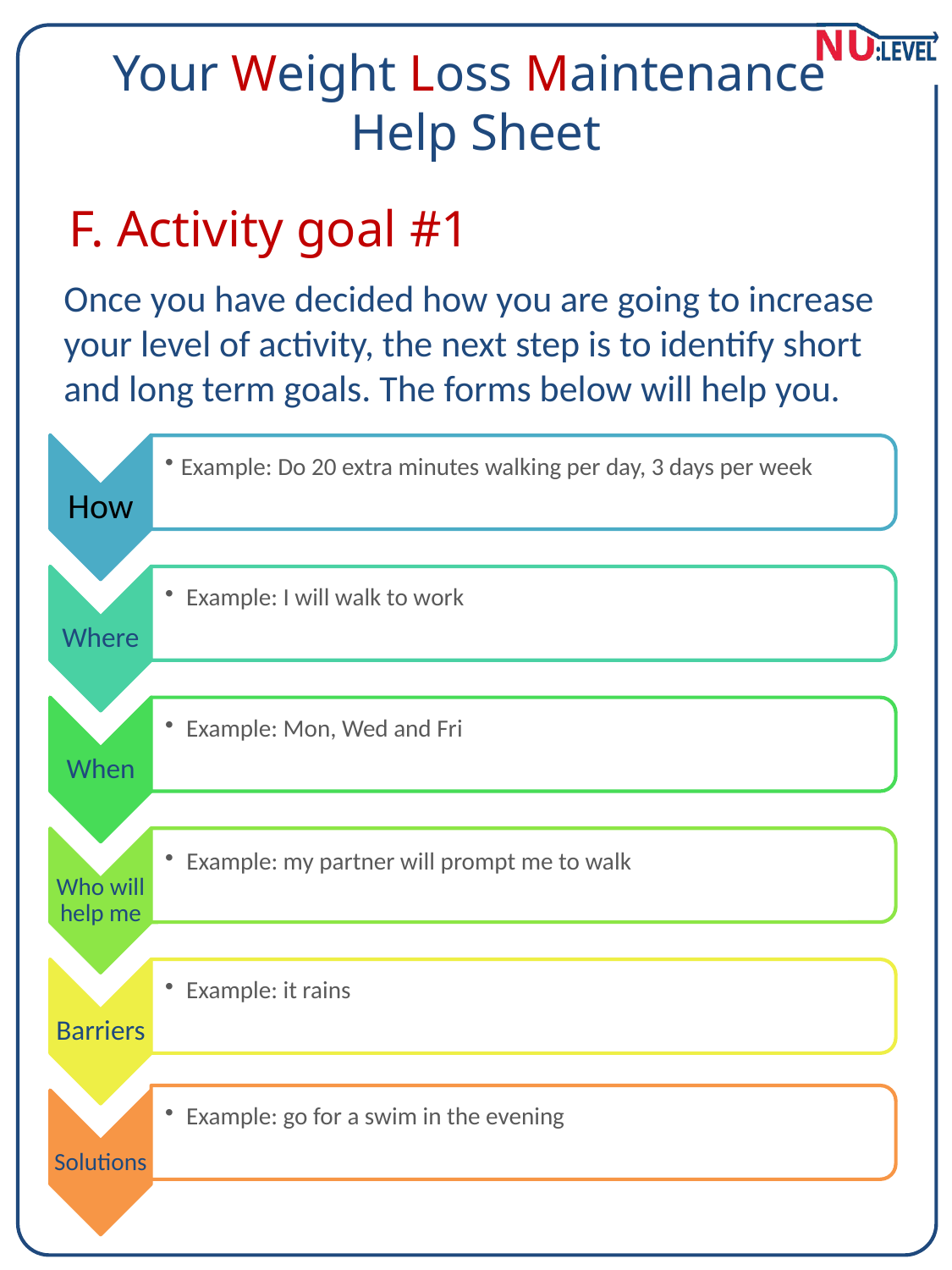

Your Weight Loss Maintenance
Help Sheet
F. Activity goal #1
Once you have decided how you are going to increase your level of activity, the next step is to identify short and long term goals. The forms below will help you.

## Slide 16
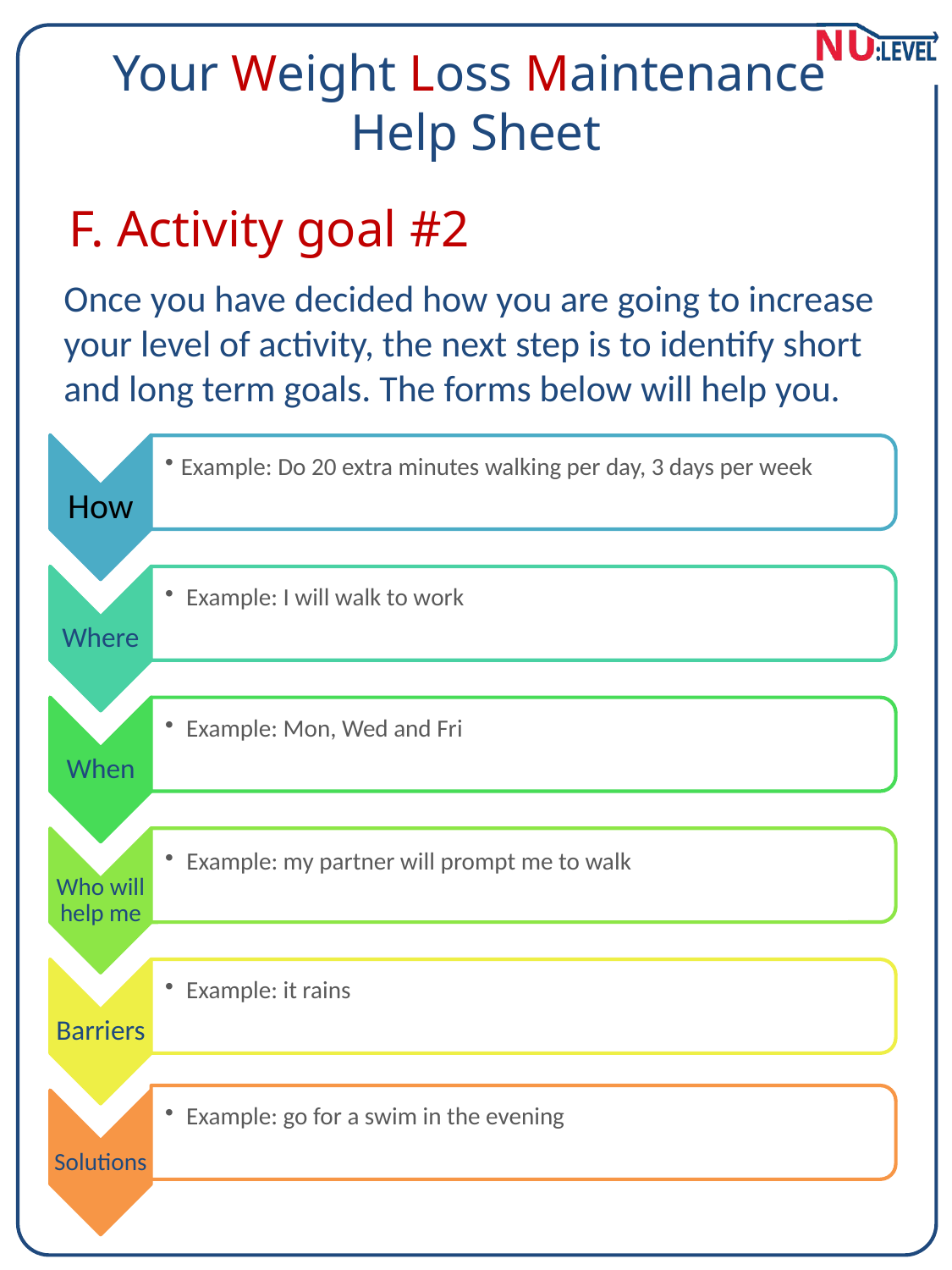

Your Weight Loss Maintenance
Help Sheet
F. Activity goal #2
Once you have decided how you are going to increase your level of activity, the next step is to identify short and long term goals. The forms below will help you.

## Slide 17
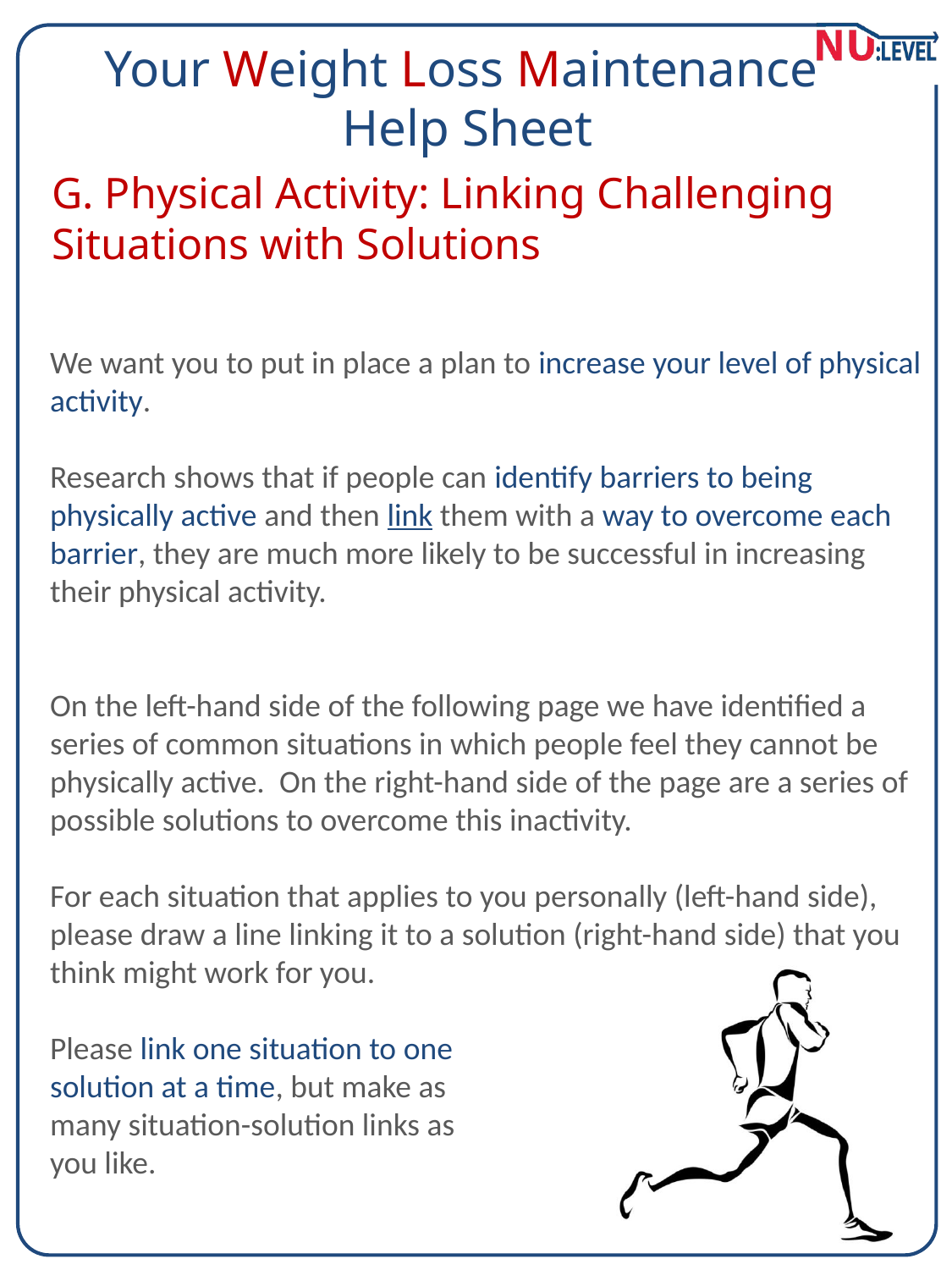

Your Weight Loss Maintenance
Help Sheet
G. Physical Activity: Linking Challenging Situations with Solutions
We want you to put in place a plan to increase your level of physical activity.
Research shows that if people can identify barriers to being physically active and then link them with a way to overcome each barrier, they are much more likely to be successful in increasing their physical activity.
On the left-hand side of the following page we have identified a series of common situations in which people feel they cannot be physically active. On the right-hand side of the page are a series of possible solutions to overcome this inactivity.
For each situation that applies to you personally (left-hand side), please draw a line linking it to a solution (right-hand side) that you think might work for you.
Please link one situation to one
solution at a time, but make as
many situation-solution links as
you like.

## Slide 18
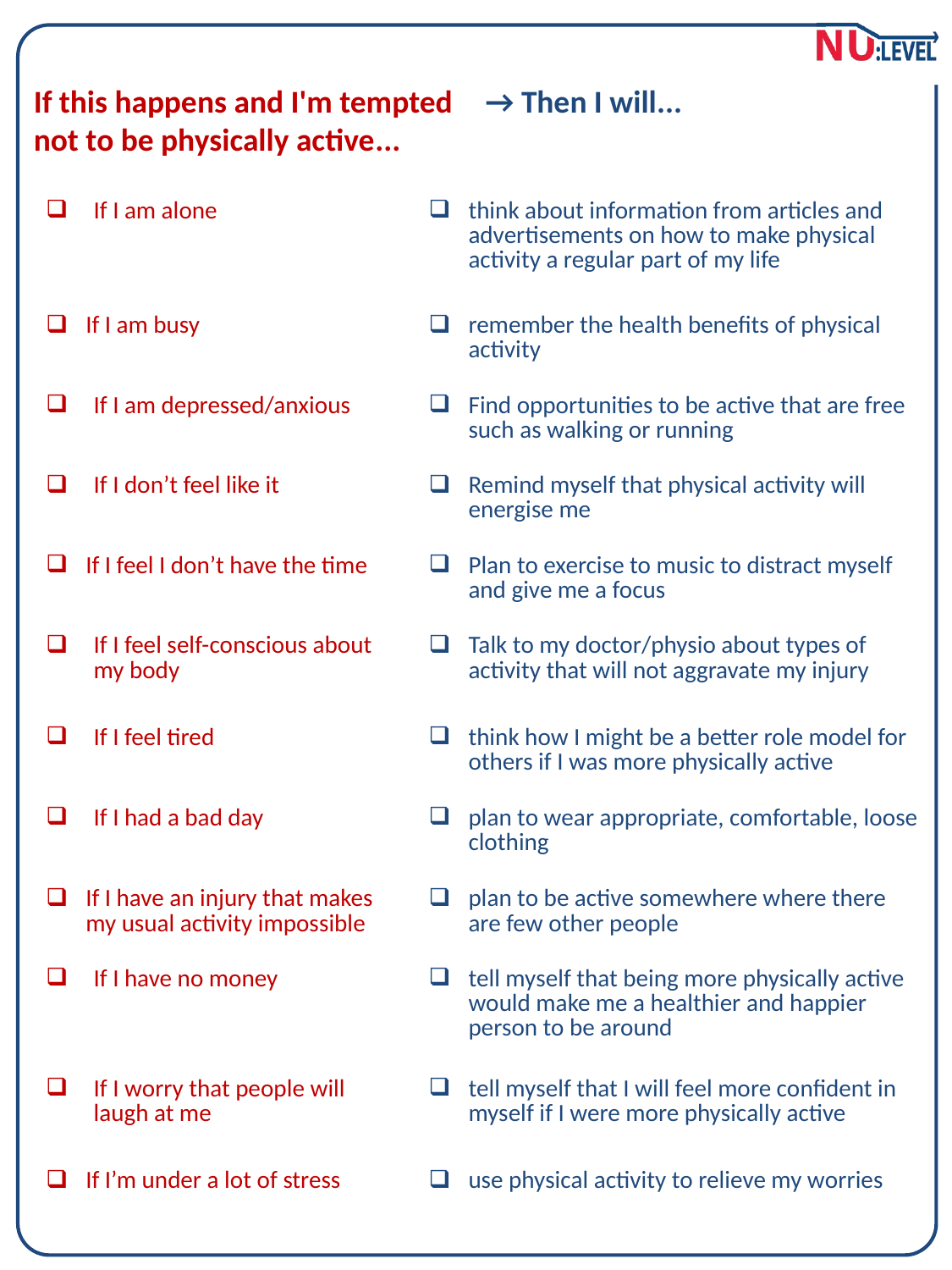

If this happens and I'm tempted not to be physically active...
 → Then I will...
| If I am alone | think about information from articles and advertisements on how to make physical activity a regular part of my life |
| --- | --- |
| If I am busy | remember the health benefits of physical activity |
| If I am depressed/anxious | Find opportunities to be active that are free such as walking or running |
| If I don’t feel like it | Remind myself that physical activity will energise me |
| If I feel I don’t have the time | Plan to exercise to music to distract myself and give me a focus |
| If I feel self-conscious about my body | Talk to my doctor/physio about types of activity that will not aggravate my injury |
| If I feel tired | think how I might be a better role model for others if I was more physically active |
| If I had a bad day | plan to wear appropriate, comfortable, loose clothing |
| If I have an injury that makes my usual activity impossible | plan to be active somewhere where there are few other people |
| If I have no money | tell myself that being more physically active would make me a healthier and happier person to be around |
| If I worry that people will laugh at me | tell myself that I will feel more confident in myself if I were more physically active |
| If I’m under a lot of stress | use physical activity to relieve my worries |

## Slide 19
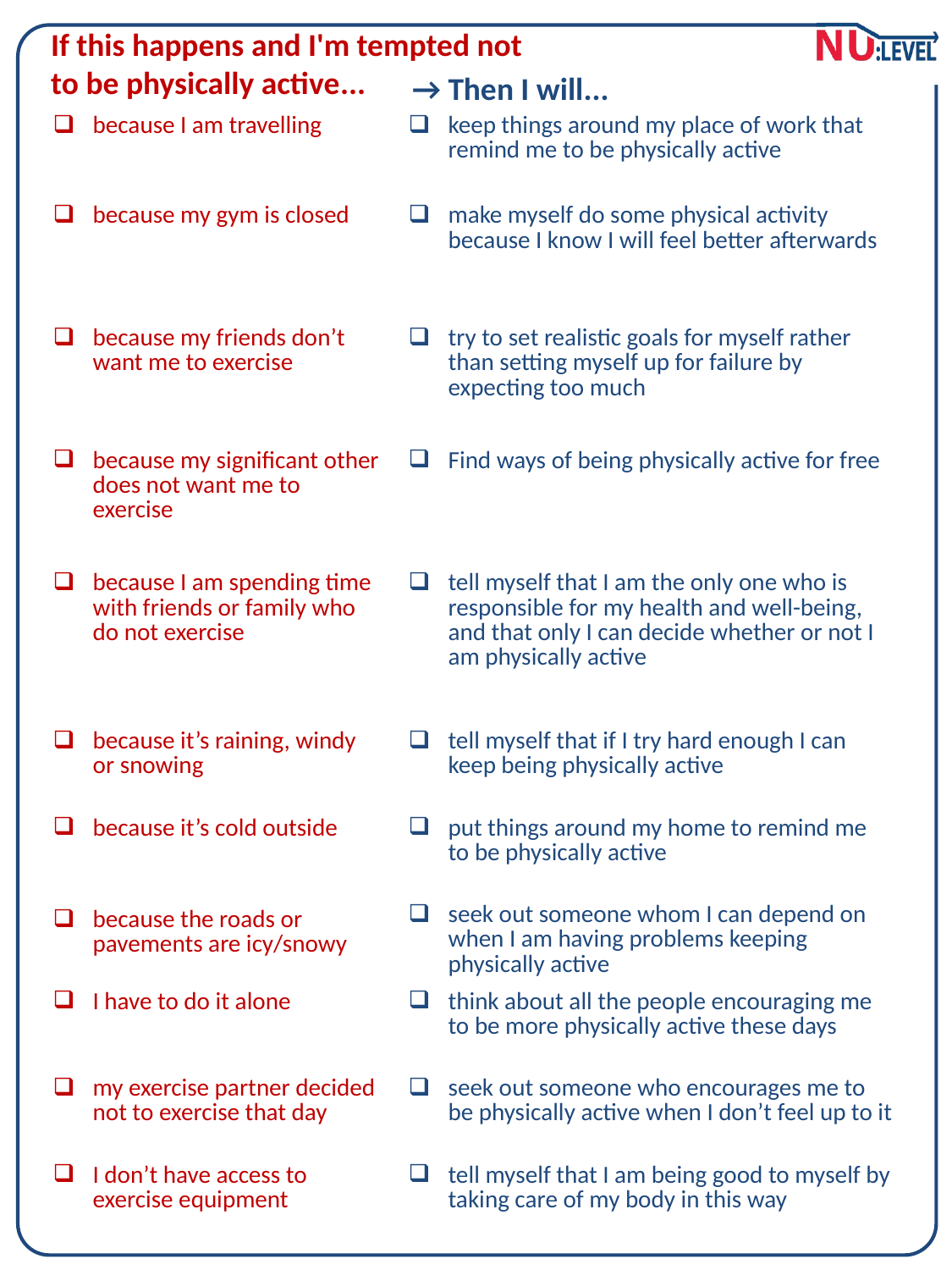

If this happens and I'm tempted not to be physically active...
 → Then I will...
| because I am travelling | keep things around my place of work that remind me to be physically active |
| --- | --- |
| because my gym is closed | make myself do some physical activity because I know I will feel better afterwards |
| because my friends don’t want me to exercise | try to set realistic goals for myself rather than setting myself up for failure by expecting too much |
| because my significant other does not want me to exercise | Find ways of being physically active for free |
| because I am spending time with friends or family who do not exercise | tell myself that I am the only one who is responsible for my health and well-being, and that only I can decide whether or not I am physically active |
| because it’s raining, windy or snowing | tell myself that if I try hard enough I can keep being physically active |
| because it’s cold outside | put things around my home to remind me to be physically active |
| because the roads or pavements are icy/snowy | seek out someone whom I can depend on when I am having problems keeping physically active |
| I have to do it alone | think about all the people encouraging me to be more physically active these days |
| my exercise partner decided not to exercise that day | seek out someone who encourages me to be physically active when I don’t feel up to it |
| I don’t have access to exercise equipment | tell myself that I am being good to myself by taking care of my body in this way |

## Slide 20
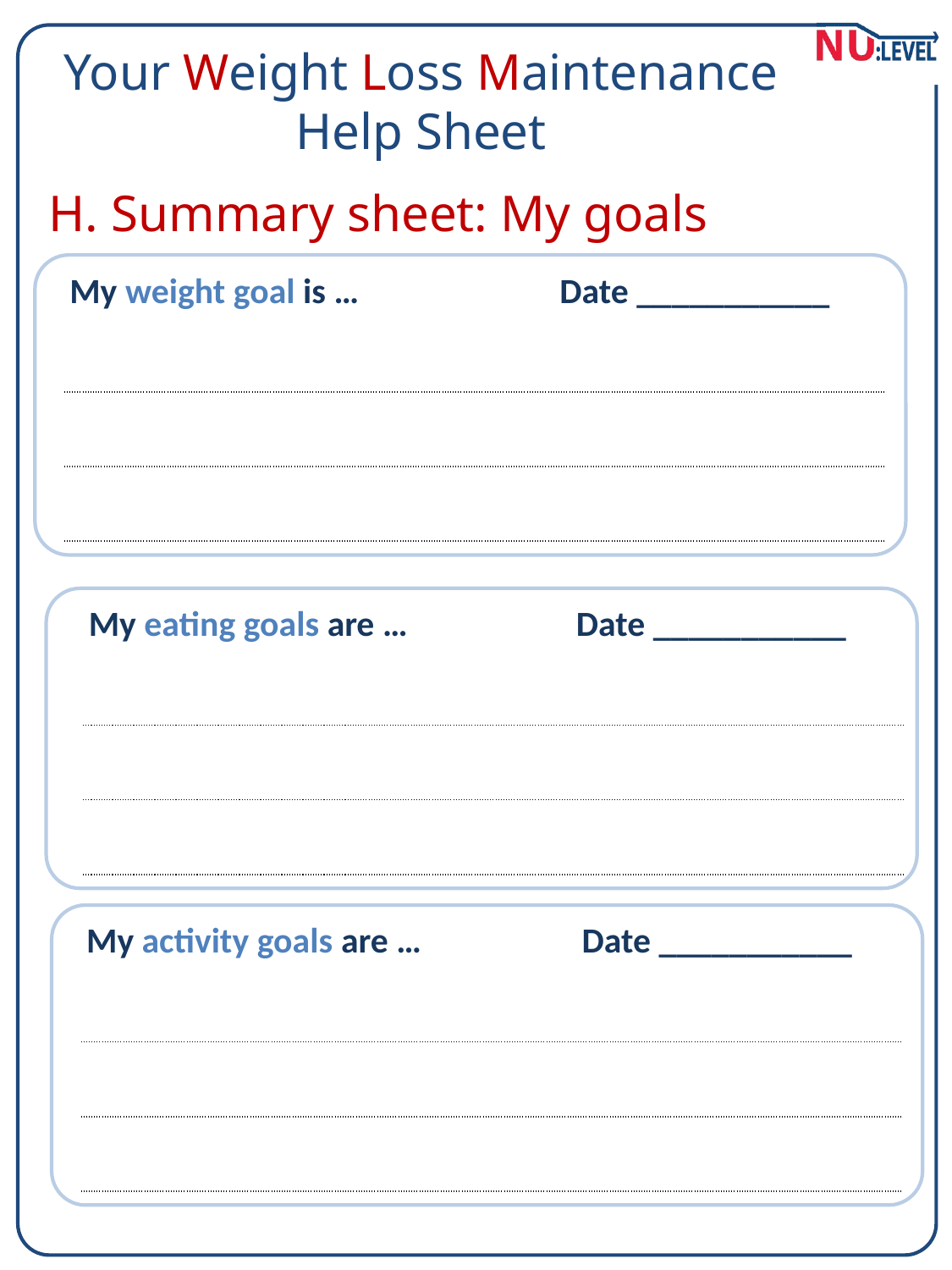

Your Weight Loss Maintenance Help Sheet
# H. Summary sheet: My goals
My weight goal is … Date ___________
| |
| --- |
| |
| |
My eating goals are … Date ___________
| |
| --- |
| |
| |
My activity goals are … Date ___________
| |
| --- |
| |
| |

## Slide 21
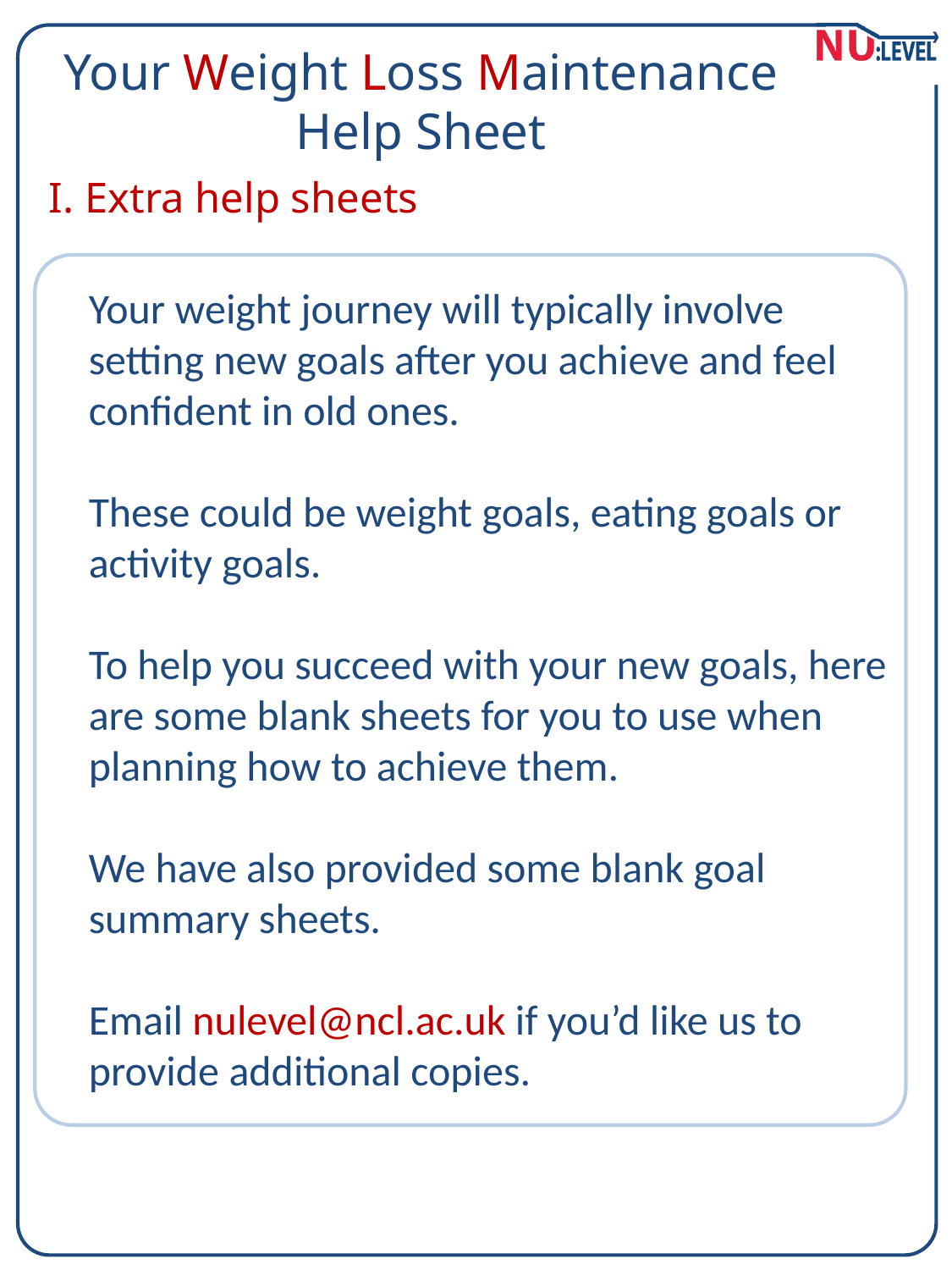

Your Weight Loss Maintenance Help Sheet
# I. Extra help sheets
Your weight journey will typically involve setting new goals after you achieve and feel confident in old ones.
These could be weight goals, eating goals or activity goals.
To help you succeed with your new goals, here are some blank sheets for you to use when planning how to achieve them.
We have also provided some blank goal summary sheets.
Email nulevel@ncl.ac.uk if you’d like us to provide additional copies.

## Slide 22
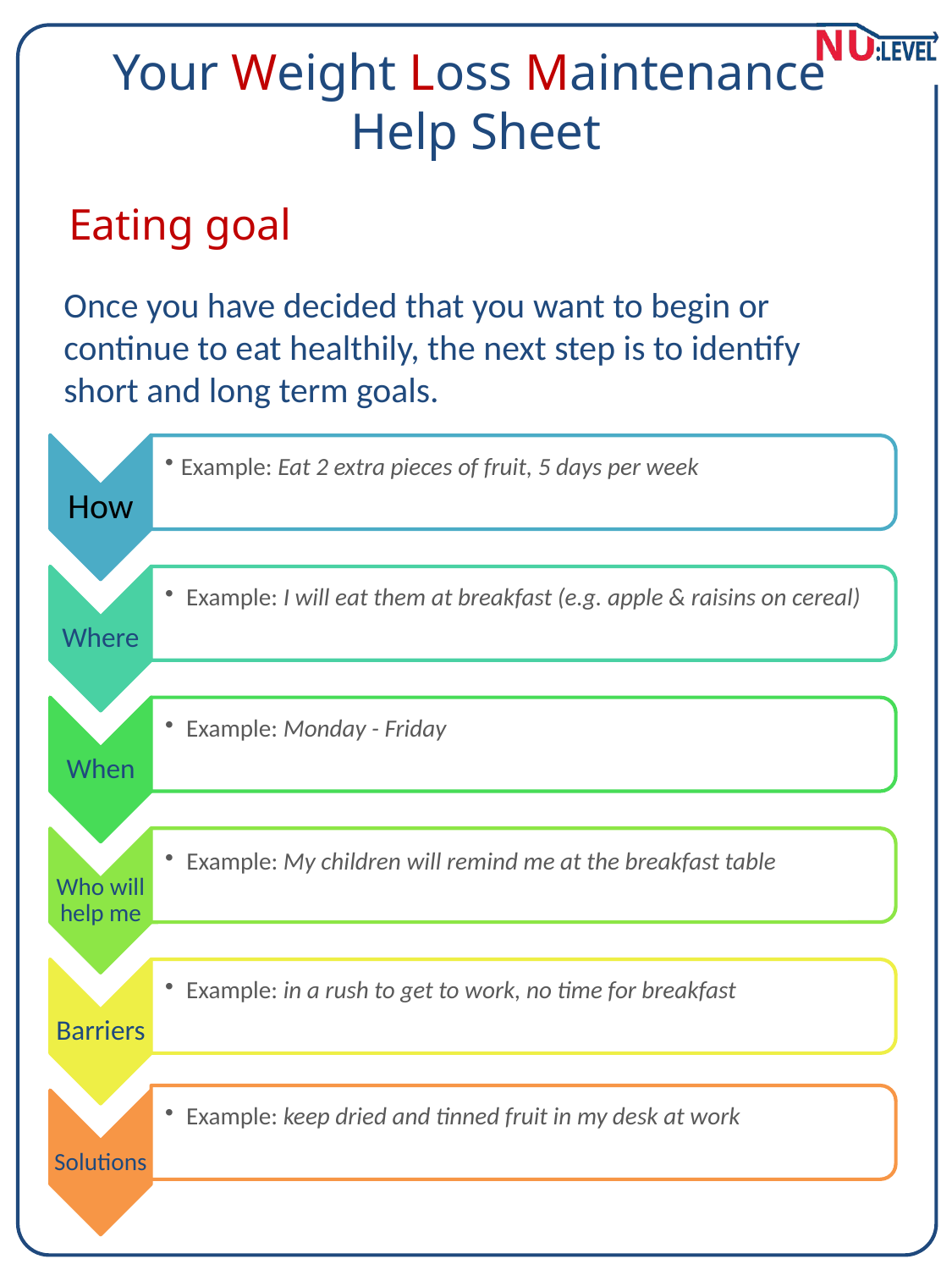

Your Weight Loss Maintenance
Help Sheet
Eating goal
Once you have decided that you want to begin or continue to eat healthily, the next step is to identify short and long term goals.

## Slide 23
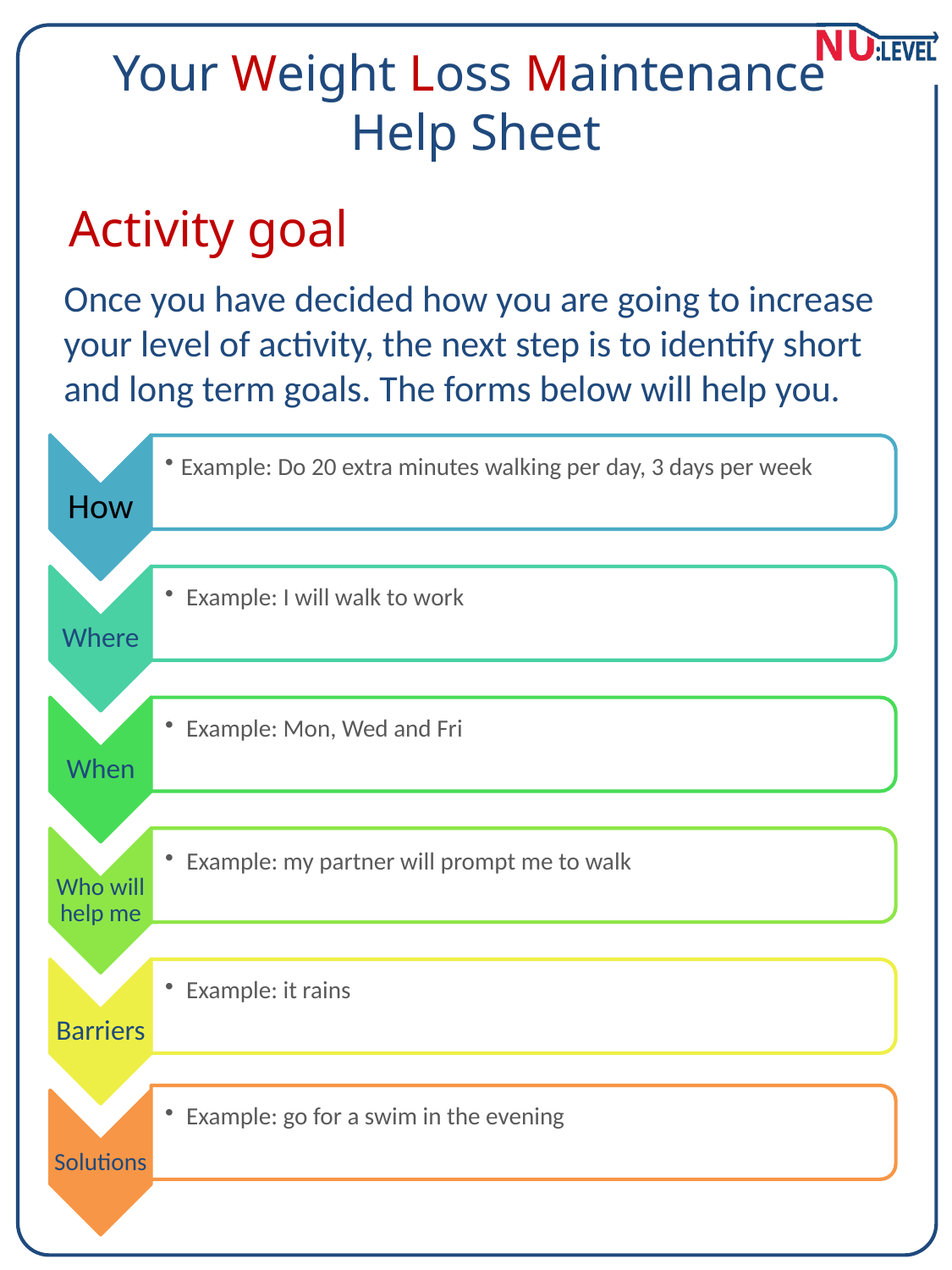

Your Weight Loss Maintenance
Help Sheet
Activity goal
Once you have decided how you are going to increase your level of activity, the next step is to identify short and long term goals. The forms below will help you.

## Slide 24
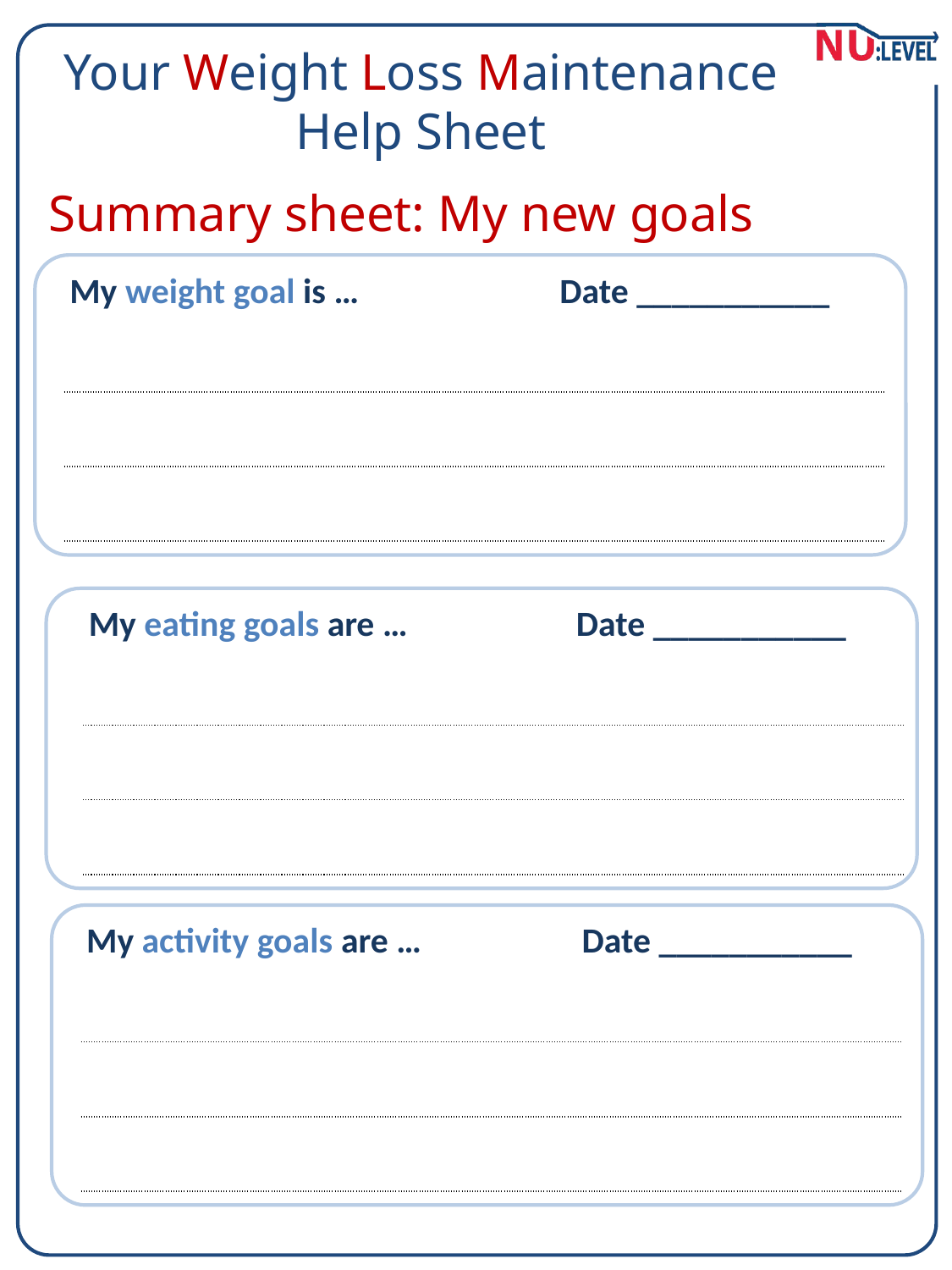

Your Weight Loss Maintenance Help Sheet
# Summary sheet: My new goals
My weight goal is … Date ___________
| |
| --- |
| |
| |
My eating goals are … Date ___________
| |
| --- |
| |
| |
My activity goals are … Date ___________
| |
| --- |
| |
| |

## Slide 25
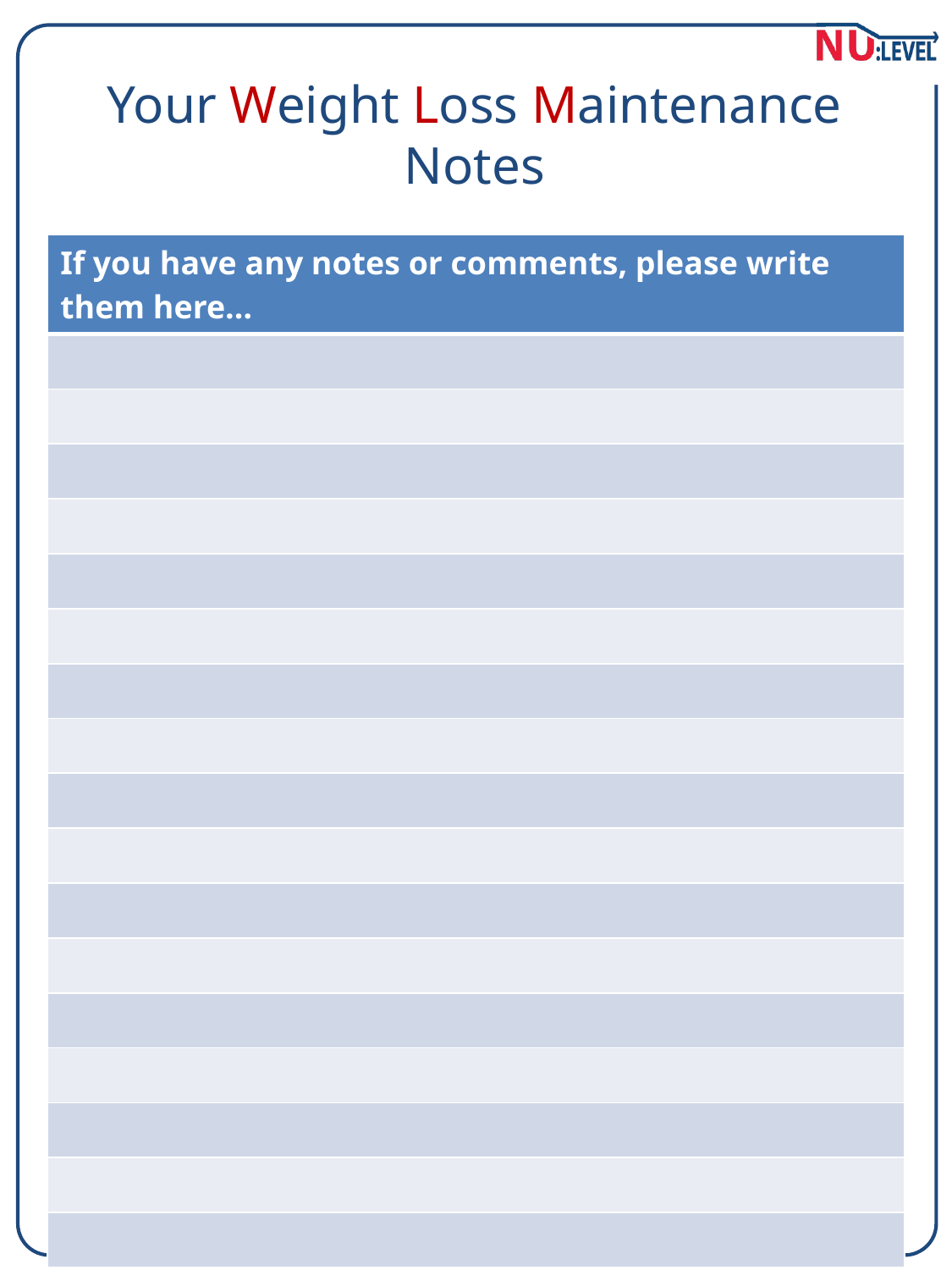

# Your Weight Loss Maintenance Notes
| If you have any notes or comments, please write them here… |
| --- |
| |
| |
| |
| |
| |
| |
| |
| |
| |
| |
| |
| |
| |
| |
| |
| |
| |
| |
| --- |
| |
| |
| |
| |
| |
| |
| |

## Slide 26
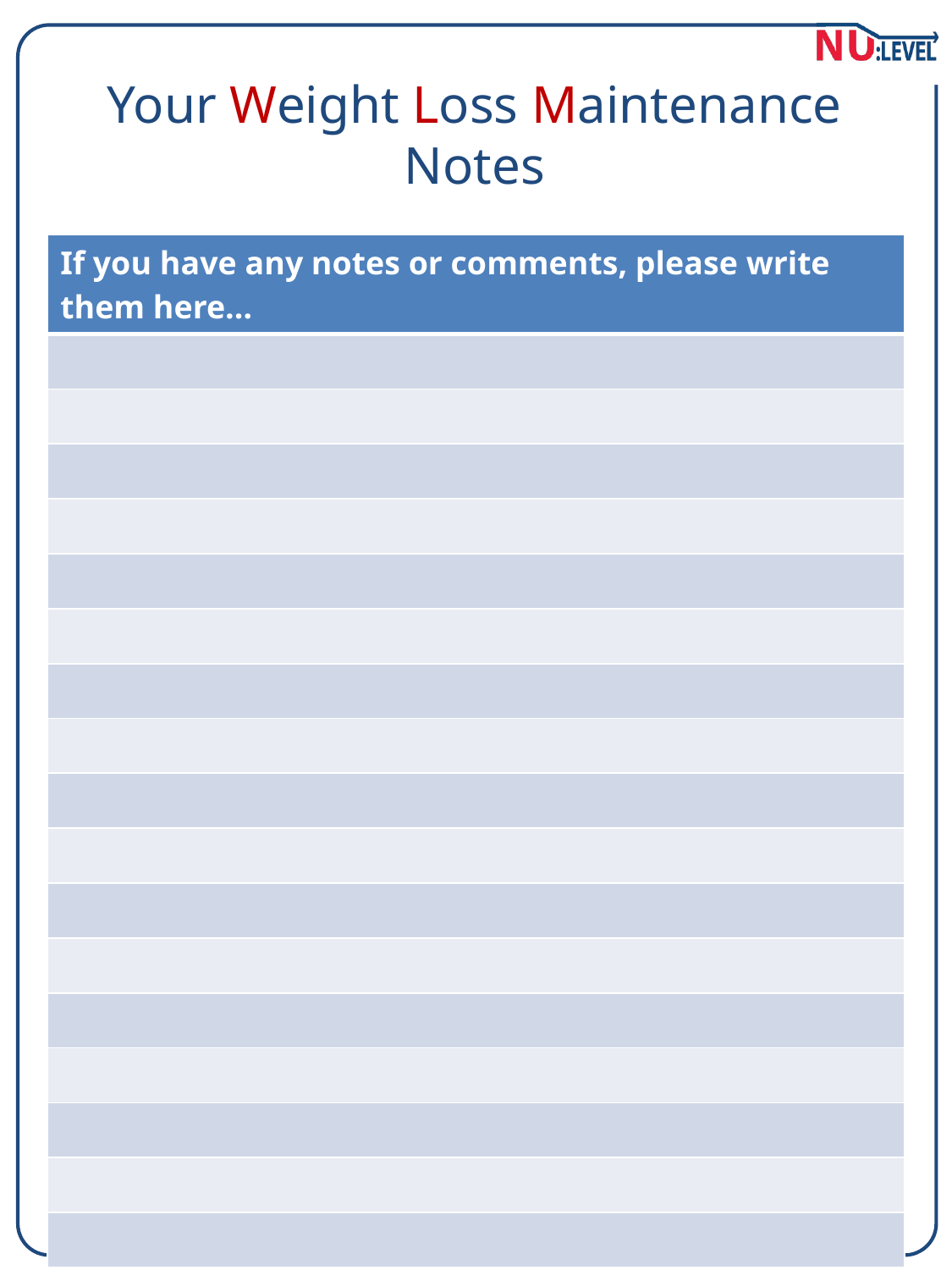

# Your Weight Loss Maintenance Notes
| If you have any notes or comments, please write them here… |
| --- |
| |
| |
| |
| |
| |
| |
| |
| |
| |
| |
| |
| |
| |
| |
| |
| |
| |
| |
| --- |
| |
| |
| |
| |
| |
| |
| |

## Slide 27
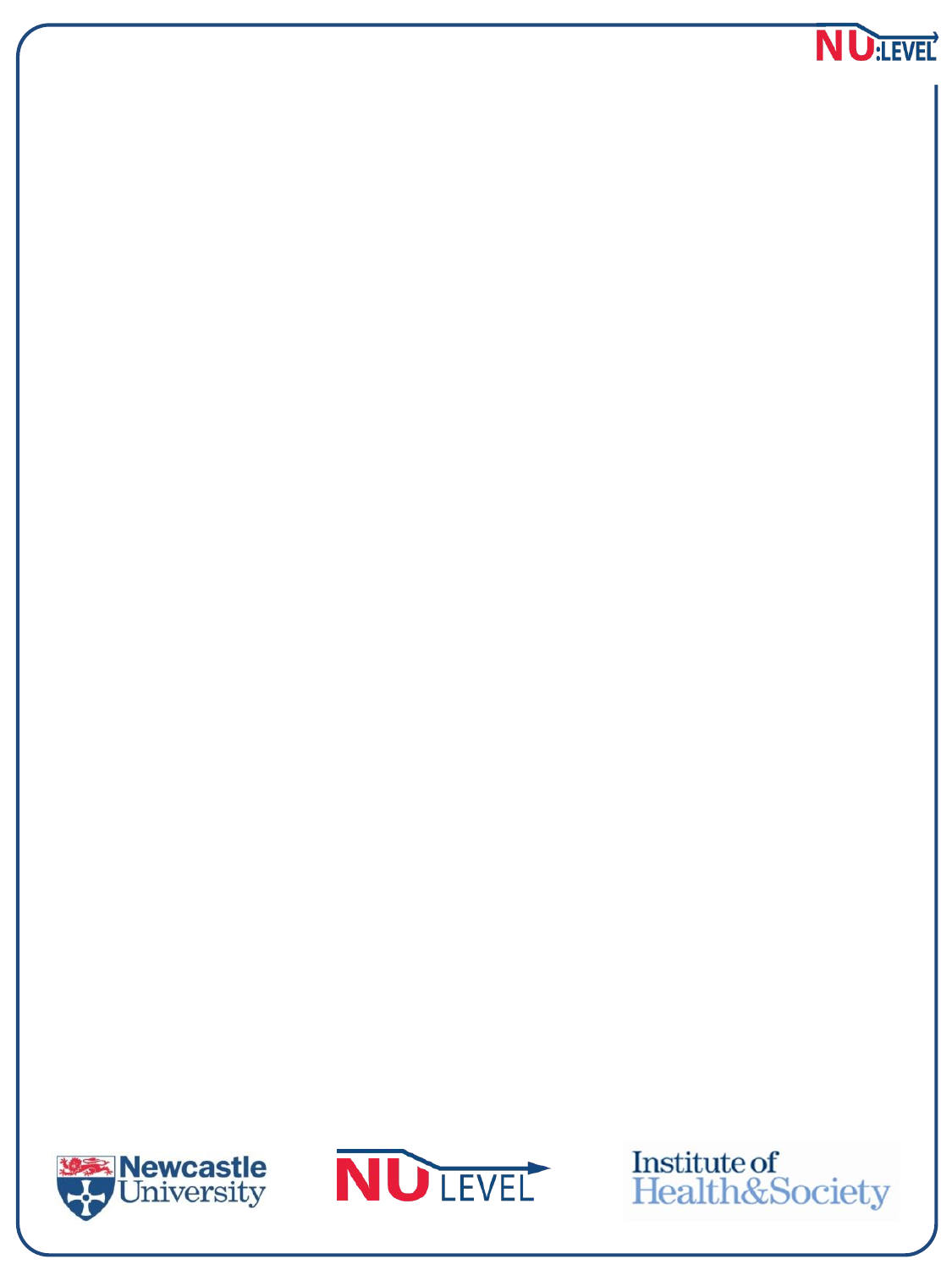

## Slide 28
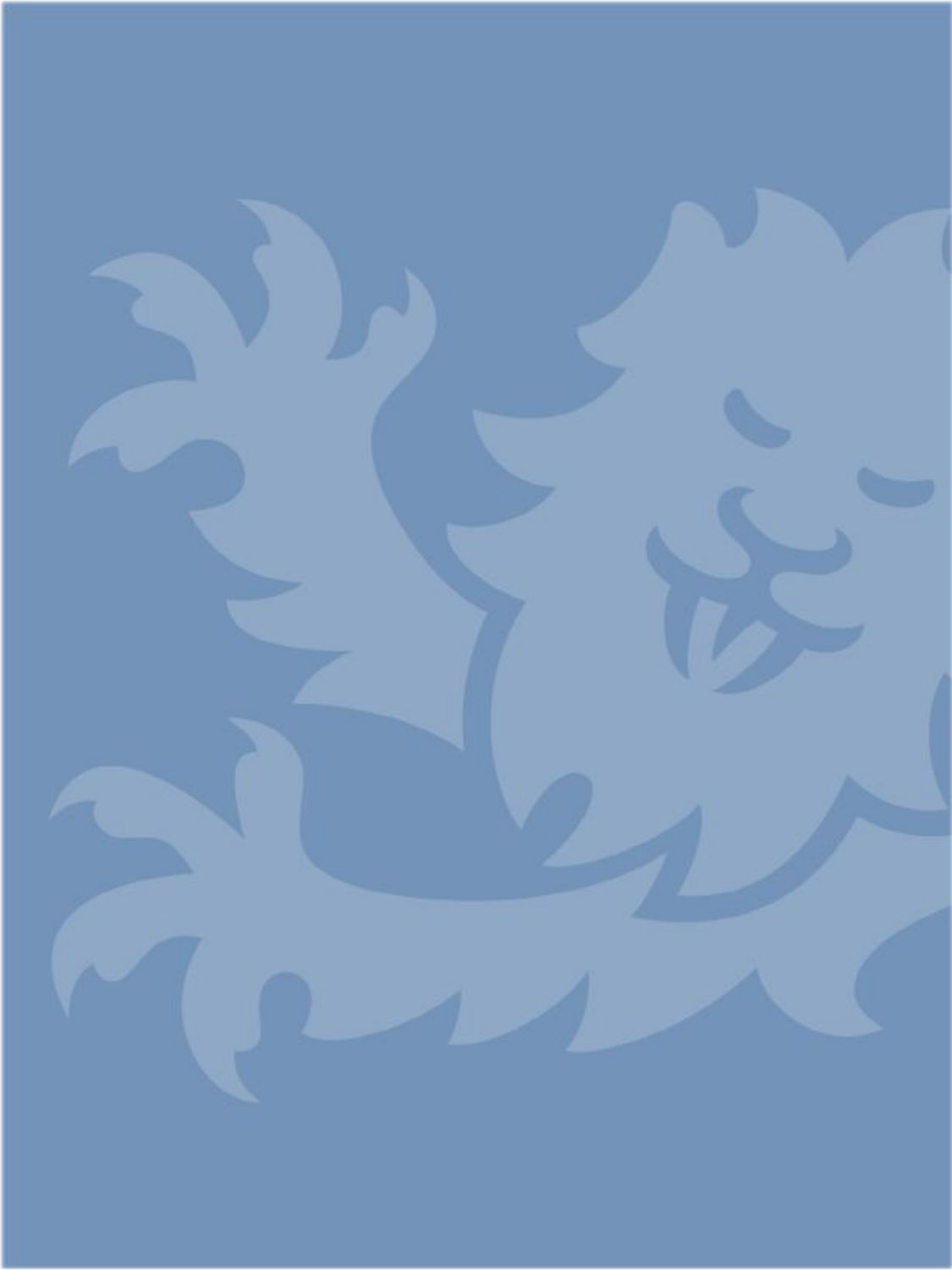

Supplement: Supplemental_Data.zip [file rhpb_a_1269233_sm0215.zip › Online supplementary material 1 SESSION MATERIALS.pptx]
